# The Effect of Conjugation with Octaarginine, a Cell-Penetrating Peptide on Antifungal Activity of Imidazoacridinone Derivative

**DOI:** 10.3390/ijms222413190

**Published:** 2021-12-07

**Authors:** Kamila Rząd, Ewa Paluszkiewicz, Damian Neubauer, Mateusz Olszewski, Katarzyna Kozłowska-Tylingo, Wojciech Kamysz, Iwona Gabriel

**Affiliations:** 1 Department of Pharmaceutical Technology and Biochemistry, Faculty of Chemistry and BioTechMed Center, Gdańsk University of Technology, 11/12 Narutowicza Str., 80-233 Gdańsk, Poland; kamila.rzad@pg.edu.pl (K.R.); ewa.paluszkiewicz@pg.edu.pl (E.P.); mateusz.olszewski@pg.edu.pl (M.O.); katarzyna.kozlowska-tylingo@pg.edu.pl (K.K.-T.); 2 Department of Inorganic Chemistry, Faculty of Pharmacy, Medical University of Gdańsk, Gen. J. Hallera 107th Avenue, 80-416 Gdańsk, Poland; damian.neubauer@gumed.edu.pl (D.N.); wojciech.kamysz@gumed.edu.pl (W.K.)

**Keywords:** cell-penetrating peptide (CPP), octaarginine, imidazoacridinone, triazoloacridinone, antifungal agents

## Abstract

Acridine cell-penetrating peptide conjugates are an extremely important family of compounds in antitumor chemotherapy. These conjugates are not so widely analysed in antimicrobial therapy, although bioactive peptides could be used as nanocarriers to smuggle antimicrobial compounds. An octaarginine conjugate of an imidazoacridinone derivative (Compound **1-R8**) synthetized by us exhibited high antifungal activity against reference and fluconazole-resistant clinical strains (MICs ≤ 4 μg mL^−1^). Our results clearly demonstrate the qualitative difference in accumulation of the mother compound and Compound **1-R8** conjugate into fungal cells. Only the latter was transported and accumulated effectively. Microscopic and flow cytometry analysis provide some evidence that the killing activity of Compound **1-R8** may be associated with a change in the permeability of the fungal cell membrane. The conjugate exhibited low cytotoxicity against human embryonic kidney (HEK-293) and human liver (HEPG2) cancer cell lines. Nevertheless, the selectivity index value of the conjugate for human pathogenic strains remained favourable and no hemolytic activity was observed. The inhibitory effect of the analysed compound on yeast topoisomerase II activity suggested its molecular target. In summary, conjugation with **R8** effectively increased imidazoacridinone derivative ability to enter the fungal cell and achieve a concentration inside the cell that resulted in a high antifungal effect.

## 1. Introduction

The discovery of amphotericin B, an antimycotic antibiotic, is considered to be one of the greatest scientific achievements of the 20th century. Due to the introduction of this drug for common use, many fungal infections that were incurable have become possible to overcome. Unfortunately, frequent and inadequate use of antifungals with a broad spectrum of activity has led to drug resistance among many species of fungi.

Acridine and acridinone derivatives are a class of compounds with a broad spectrum of biological activity and are very interesting for scientists. Concerning their mode of action, these compounds are able to render DNA damage, disrupt DNA repair and replication, inhibit topoisomerase I and II enzymes [1], and induce cell death [2]. A lot of derivatives have been obtained with antitumor, antibacterial, and antifungal activity [3,4,5,6,7]. As far as antimicrobial activity is concerned in many cases, their application is limited and excluded because of transport inefficiency. As described previously for imidazoacridinone **C-1311** and its 9 derivatives, only three that entered fungal cells showed phototoxic antifungal activity (**C-1330**, **C-1415** and **C-1558**) [8]. Despite the high efficiency of **C-1311** as an anticancer compound [9,10] (it was in phase II clinical trials for the treatment of women with metastatic breast cancer [11]), it, among other things, intercalates into DNA [12,13] and exhibited human topoisomerase inhibition activity [14], it was unable to accumulate in *Candida albicans* cells and no antifungal activity was observed [8]. The results obtained for capridine β (1-nitro-9-aminoacridine derivative) indicate that not only efficient accumulation, but also the biotransformation into a metabolite that can affect fungal topoisomerase II are important with respect to antifungal activity [15].

With the current massive increases in drug-resistant microbial infection, new derivatives that exhibit multiple modes of action may be extremely important. Acridines, acridones and their derivatives are considered to be photoantimicrobials, with multiple targeted modes of actions [8], relatively immune to individual resistance mechanisms [16]. Moreover, those compounds’ antimicrobial activity may also be associated with an inhibitory effect on microbial gyrase or topoisomerase. DNA topoisomerases/gyrases are essential enzymes that catalyse topological changes in DNA and therefore also play key roles in the proper functioning of the cell during its divisions. These enzymes are the targets of important anticancer and antibacterial drugs. Human type IIA topoisomerases are the targets of the widely used anticancer agents such as etoposide, anthracyclines and mitoxantrone [17]. Bacterial type II topoisomerases (gyrase and Topo IV) are the targets of quinolones and aminocoumarin antibiotics [18], whereas eukaryotic type IB topoisomerases (Top1) are targeted by camptothecin and its derivatives and novel noncamptothecins in clinical development (indenoisoquinolines and ARC-111) [19]. Prokaryotic topoisomerases are good targets for antibacterial chemotherapy, firstly, because they are essential in all bacteria for replication and cell division, secondly, because an accumulation of cleavage complexes has a bactericidal effect, and thirdly, because targeting bacterial type II topoisomerases is not poisonous for human enzymes [18,19,20]. Functional topoisomerase II is also essential for the growth of yeast [21,22]. The differences between fungal and human topoisomerases have also led to the suggestion that this class of enzymes may be potential targets for the development of novel antifungal agents [23,24]. Our previously published data indicate that yeast topoisomerase II’s relaxation ability has been effectively inhibited by an acridine derivative with antifungal activity [7].

Amino acid or peptide analogues of acridines (PACs, peptide-acridine conjugates) constitute an extremely important family of compounds in antitumor chemotherapy [25]. That conjugates of acridines or acridinones in antimicrobial therapy are not so widely analysed, although bioactive peptides could be used as nanocarriers to smuggle antimicrobial compounds into yeast or bacterial cells to reach their intracellular targets. In recent years, compounds with high antibacterial activity and the ability to exhibit dual mechanisms of action have been described. Acridine conjugates with short cationic bioactive peptide activity results from the disruption of the cell membrane by pore formation on one side and intracellular DNA binding as the second target [26]. The double targets reduce the risk of developing resistance. It was also demonstrated that PACs are able to bind RNA aptamers with high discrimination over DNA. This ability confers them individual character and distinguishes them from intercalating acridine binders. PAC compounds with a substitution pattern of the peptide localized at positions 4 and 9 of the tricyclic ring are selective RNA binders. The mechanism of binding is called ‘threading intercalation’. This involves stabilising interactions between substituents and functionality present in the grooves of the duplex or bulged looped nucleotides found adjacent to the intercalation site [27].

Interestingly, only few examples of amino acid or peptide analogues of acridines with antifungal activity have been described. Recent studies indicated that branched, lysine-rich acridine conjugates show strong antifungal and antibiofilm activity and no hemolytic effects [28]. The introduction of lysine moiety into the acridine core of known anticancer compound **C-1748** (also known as Capridine β [29]) resulted in obtaining a derivative that targets yeast topoisomerase II and which is even active against fluconazole-resistant *C. albicans* strains with MIC values in the range of 16–64 μg mL^−1^ [7].

**Figure 1:**
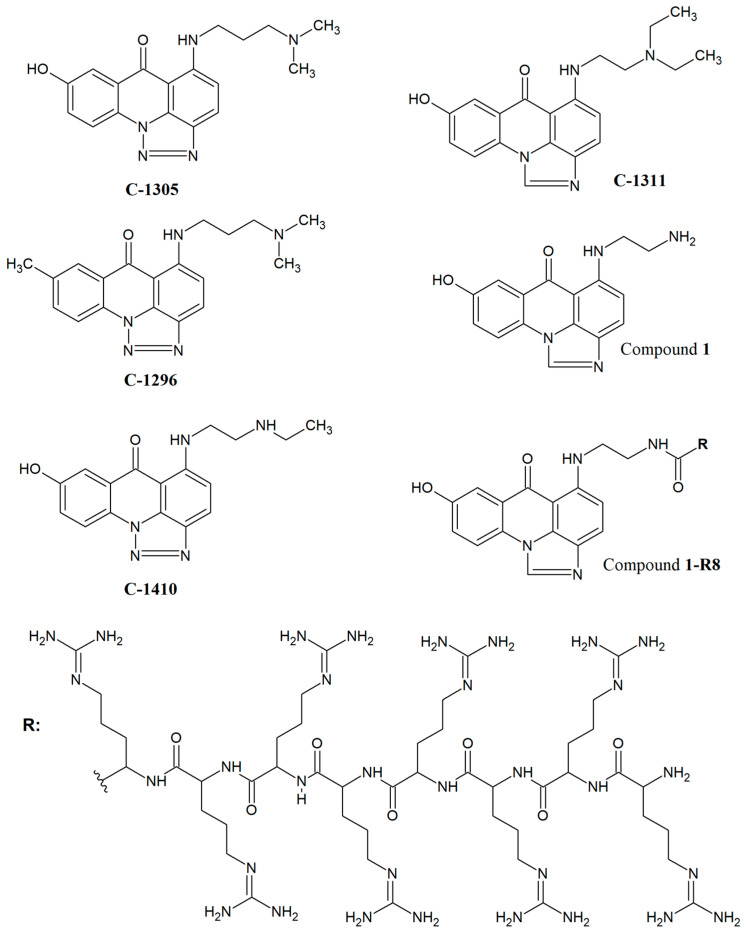
The structures of **C-1305**, **C-1311** and their derivatives that were synthesized in this study.

Emerging from previous studies performed for acridines and acridinones as potential antimicrobial agents [3,4,5,6,7,8], we have decided to analyse the antifungal activity of imidazo- and triazoloacridinone derivatives. Hence, in this paper, we report the synthesis and biological properties of **C-1311** and **C-1305** analogues (Figure 1) and the ability of bioactive peptide octaarginine to smuggle **C-1311** imidazoacridinone scaffold into fungal cells. As acridine and acridinone derivatives with anticancer activity are reported to be effective human topoisomerase I and II inhibitors (e.g., mitoxantrone [30], **C-1311** and **C-1305** [14,31], m-AMSA [17] or 9-aminoacridine derivatives [32]), we have also analysed the inhibitory effect of selected derivatives on yeast topoisomerase II activity (yTOPO II).

## 2. Results and Discussion

### 2.1. Synthesis

#### 2.1.1. Synthesis of Triazoloacridinone and Imidazoacridinone Derivatives

We have synthesized compounds that are structural analogues of triazoloacridinone **C-1305** [S-[[3-(dimethylamino)propyl]amino]-8-hydroxy-6H-v-triazolo[4,5,1-de]acridin-6-one] and imidazoacridinone **C-1311** [5-((2-(diethylamino)ethyl)amino)-8-hydroxy-6H-imidazo[4,5,1-de]acridin-6-one] (Figure 1). The 8-substituted 5-[(aminoalkyl)amino]-6H-v-triazolo[4,5,l-de]-acridin-6-ones (**C-1305**, **C-1296** and **C-1410**) presented in Figure 1 were prepared as monohydrochlorides according to the previously reported procedure [33]. The strategy of the synthesis is presented in the Supplementary Materials in Figure S1.

The second group of synthesized compounds, imidazoacridinone derivatives (**C-1311** and Compound **1**) presented in Figure 1 were synthesized, starting from 1-chloro-7-hydroxy-4-nitro-9(10H)-acridinone through to the substitution of aliphatic amines and reduction and cyclization of the resulting derivatives. This method of synthesis was described previously [34,35]. The strategy of the synthesis is presented in the Supplementary Materials in Figure S2. Compound **1** was used as a starting material for the synthesis of derivative Compound **1-R8**.

#### 2.1.2. Peptide Synthesis

Octaarginine (**R8**) was purchased from Sigma-Aldrich (Sigma Aldrich, St. Louis, MO, USA). **R8** analogue of Compound **1** (Compound **1-R8**) was synthetized manually by the solid-phase Fmoc/tBu method as described in Section 3. The strategy of the synthesis is presented in the Supplementary Materials in Figure S3. Purification was performed by RP-HPLC and the identity of Compound **1-R8** was confirmed with ESI-MS (Section 3). The results of ESI-MS analysis are presented in Supplementary Materials in Table S1. Analytical HPLC analysis of Compound **1-R8** conjugate purity is presented in Supplementary Materials in Figure S4.

As Compound **1** has two amino groups (2-aminoethylamino; AEA, moiety that is attached at position 5) that can react with Fmoc-L-Arg(Pbf)-OH, additional NMR analyses were performed to confirm that the arginine residue is conjugated with the primary amino group of AEA. To facilitate the analysis of NMR spectra, the Compound **1-R** conjugate was used instead of Compound **1-R8**. The structure of the Compound **1-R** conjugate was determined with ^1^H NMR and COSY analysis (Supplementary Materials). Chemical shifts of protons of AEA, 3.51 and 3.41, found for one of methylene groups and 3.58 for another are typical for those attached to the nitrogen atoms of the secondary amine and amide. These chemical shifts confirmed that the arginine residue is conjugated with the primary amino group of AEA.

### 2.2. Susceptibility Testing of Novel Compounds against Fungal Strains

The starting compounds and derivatives were tested for their in vitro antifungal activity against five reference strains. The most active compound was tested against six *C. albicans* clinical isolates, sensitive (B3, Gu4 and F2) and resistant (B4, Gu5 and F5) to fluconazole [36,37]. Minimal inhibitory concentrations (MICs) of the studied compounds determined by the microplate serial dilution method are shown in Table 1 and Table 2.

As reported in Table 1, no antifungal activity was observed for all analysed imidazoacridinone (**C-1311** and Compound **1**) and triazoloacridinone derivatives (**C-1305**, **C-1296**, **C-1410**), except for the octaarginine conjugate of Compound **1**. The activity of that conjugate was even higher than amphotericin B for *C. krusei* and *C. parapsilosis*. The FLU-resistant clinical B4, Gu5 and F5 strains were sensitive to conjugate Compound **1-R8** at the same level as the FLU-sensitive isolates—B3, Gu4, and F2 (Table 2). The analysed *C. albicans* strains are resistant to fluconazole due to the FLU-induced overexpression of Cdr1p/Cdr2p drug efflux pumps, as well as Mdr1p membrane transport protein of the major facilitator superfamily [36,37]. Thus, our results indicate that the conjugate is not a good substrate for Cdr1p/Cdr2p and Mdr1p.

One of the key parameters for the success of antifungal drug molecules is the ability to enter fungal cells. This is related to the compounds’ physicochemical parameters. In order to determine lipophilicity, in silico calculation was carried out [38]. We have determined Log P_o/w_, which is a very valuable parameter to estimate the ability of a compound to move/cross from the aqueous phase to lipid phase. This allows for the ability of compounds to cross the cell membranes estimation. As can be seen in Table 3, the antifungal activity of the imidazoacridinone (**C-1311** and Compound **1**) and triazoloacridinone derivatives (**C-1305**, **C-1296**, **C-1410**) analysed by us, as well as those previously published (**C-1330**, **C-1415**, **C-1558**) [8], seemed to be correlated with their lipophilicity, except for the octaarginine conjugate of Compound **1**.

Our results indicate that simple diffusion into the cell interior is not responsible for the high antifungal activity of Compound **1-R8**. We have carried out a more in-depth analysis of its mechanism of action to evaluate whether bioactive peptide **R8** could be used as a nanocarrier to smuggle Compound **1** into fungal cells to reach its intracellular targets.

### 2.3. Molecular Mechanism of Antifungal Activity

#### 2.3.1. Killing Activity of Compound **1-R8**

**Figure 2:**
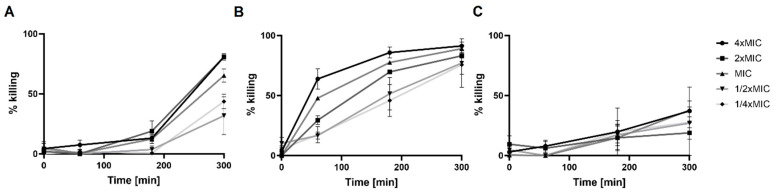
Killing kinetics of Compound **1-R8** (**A**), amphotericin B (**B**), and fluconazole (**C**) on *C. albicans* ATCC 10231 in RPMI medium. (●) 4 × MIC of Compound **1-R8**, amphotericin B and fluconazole (4, 2 and 32 μg mL^−1^, respectively). (▪) 2 × MIC, (▲) MIC, (▼) 1/2 × MIC, and (♦) 1/4 × MIC of analysed compounds. The experiments were performed in at least three replicates (% killing ± SEM).

To establish the possible mode of action for Compound **1-R8**, we have analysed the killing activity and determined minimal fungicidal concentrations (MFCs) (Table 4). Additionally, killing kinetics analysis against pathogenic *C. albicans* ATCC 10231 was performed (Figure 2).

The killing kinetics against pathogenic *C. albicans* were evaluated at different drug concentrations corresponding to 4 × MIC, 2 × MIC, MIC, 1/2 × MIC and 1/4 × MIC. A time- and dose-dependent candidacidal activity of Compound **1-R8** was observed (Figure 2).

Amphotericin B showed much faster killing kinetics than Compound **1-R8** (Figure 2). As shown in Figure 2B, drug presence caused about 60% mortality of the microbial population within 1 h at 4 × MIC (2 μg mL^−1^), whereas for Compound **1-R8** conjugate, a much longer incubation time is needed to kill the fungal cells. As expected, the effect of fluconazole was fungistatic (Figure 2C).

#### 2.3.2. Compound **1** and Compound **1-R8** Accumulation in Fungal Cells

**Figure 3:**
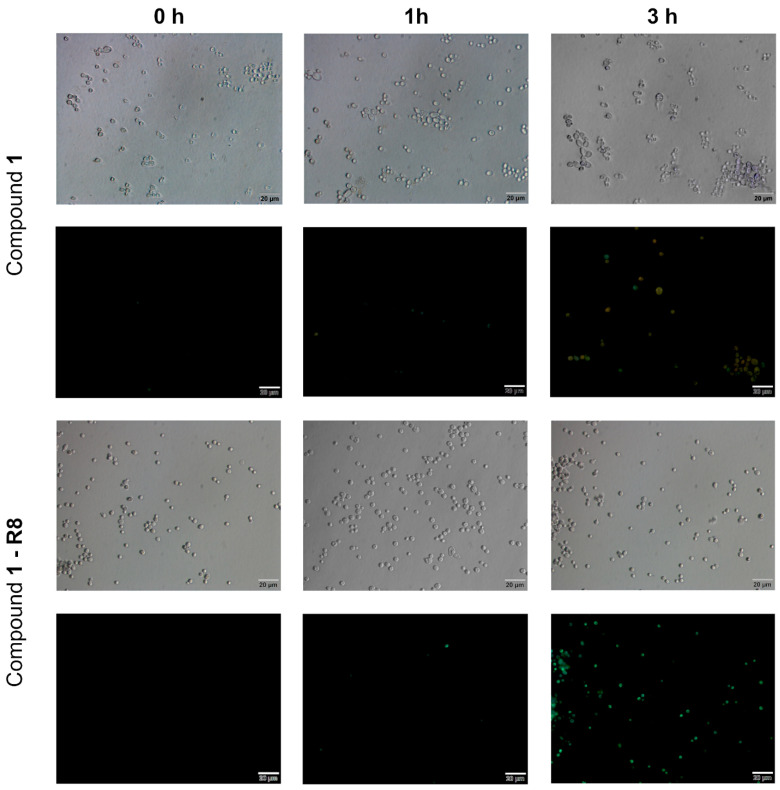
Fluorescence microscopic analysis of uptake and accumulation of Compound **1**; Compound **1-R8** in *C. albicans* cells ATCC 10231. Cells were suspended in phosphate-buffered saline and incubated in the presence of fluorescent probes at 8 × MFC of Compound **1-R8** concentration (256 μg mL^−1^) for appropriate period of time. Scale bars correspond to 20 μm.

The fluorescent properties of imidazoacridinone derivatives (Compound **1** and Compound **1-R8**) were used to analyse the ability to penetrate into the fungal cell (Figure 3).

Figure 3 clearly demonstrates the qualitative difference in the accumulation of the analysed compounds into fungal cells. Only Compound **1-R8** was transported to and accumulated in *C. albicans* ATCC 10231 cells effectively, while no significant accumulation was detected for Compound **1**. The uptake was time- (as evidenced in Figure 3) and concentration-dependent (data not shown). These results provide a rational explanation for the lack of antifungal activity of the latter. However, it is possible that for that compound, the elimination rate is greater than penetration efficiency. Conjugation of Compound **1** with **R8** effectively increased its ability to enter a fungal cell and achieve a concentration inside the cell that results in an antifungal effect.

As was reported previously, carboxyfluorescein-labeled nonaarginine (CF-R9) uptake into the model yeast *S. cerevisiae* proceeded according to the mechanism of energy-dependent endocytosis, and the internalization of CF-R9 reached a saturation point after 15 min of incubation with the peptide [39]. In our studies with *C. albicans* cells, an intense green signal was observed after a much longer period of time (Figure 3).

**Figure 4:**
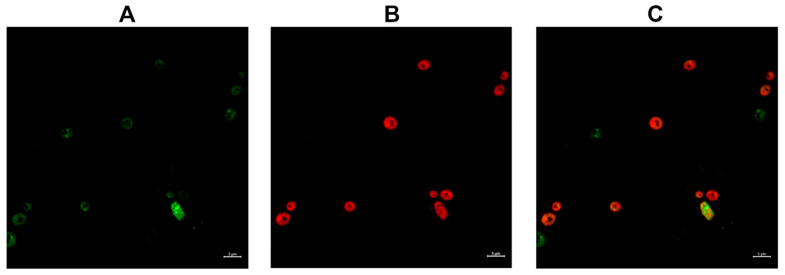
Confocal microscopic analysis of propidium-iodide (PI) uptake into *C. albicans* ATCC 10231 cells treated for 1 h with Compound **1-R8** (8 × MFC). (**A**) The green, fluorescent signal associated with the accumulation of Compound **1-R8** (**B**) the red fluorescent signal associated with PI uptake and (**C**) merged signals. Scale bars correspond to 5 μm.

To better understand whether the killing activity of Compound **1-R8** may be associated with a change in the permeability of the cell membrane *C. albicans* ATCC 10231 cells were incubated with Compound **1-R8** (8 × MFC) for 1 h; then, fungal cells were subsequently incubated with 1 μg mL^−1^ propidium iodide (PI) for 30 min. Cells were harvested by centrifugation and washed twice with PBS. The fluorescent dye PI takes into account membrane permeability as a parameter to differentiate between live and dead cells [40]. Live cells are impermeable to PI, and hence an increase in PI staining suggests loss of viability associated with a change in the integrity of the cell membrane. Although recently published data suggested that, for *S. cerevisiae* cells, it is possible for PI-exposed yeast cells to be red but not dead, the subpopulation of cells exhibiting the ability to repair was estimated at approximately 7% [41]. It is still a well-established and rapid method for monitoring membrane damage. PI-negative and PI-positive cells were observed by confocal microscopy (Figure 4).

Figure 4 clearly demonstrates that the incubation of *C. albicans* ATCC 10231 with Compound **1-R8** resulted in PI-positive cells. Thus, the effect of octaarginine conjugate on the fungal membrane might be associated with a change in its permeability or damage, as well as the loss of fungal cell viability.

**Figure 5:**
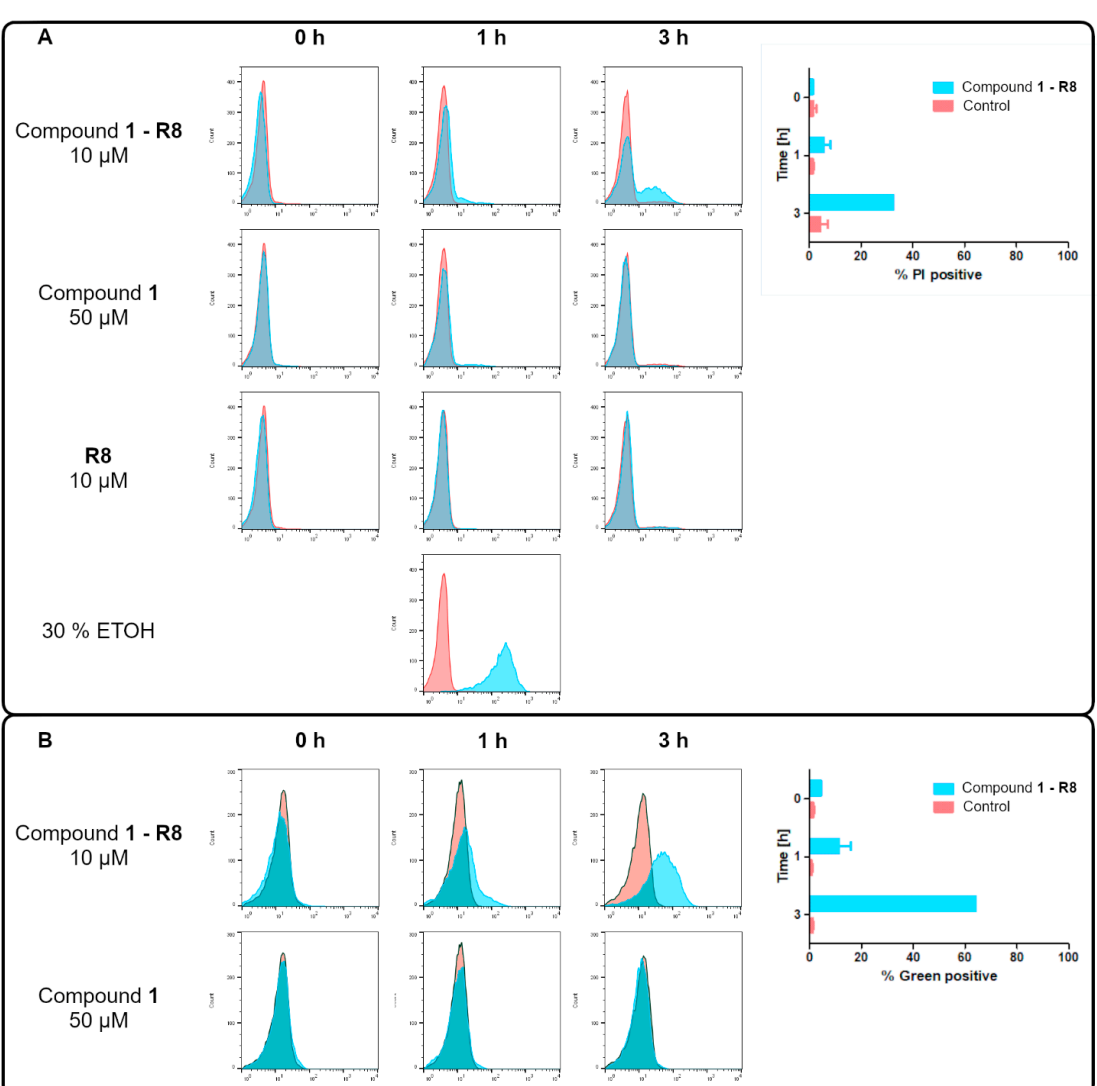
Representative flow cytometric analysis of *C. albicans* ATCC 10231 cells untreated (red) and treated (blue) with Compound **1-R8** at concentration corresponding to 1/2 × MFC (10 μM), Compound 1 (50 μM) for 0, 1, and 3 h and 30% EtOH for 1 h (positive control) in the presence of 1 μg mL^−1^ of PI (final concentration). (**A**) Red fluorescence of PI and (**B**) green fluorescence associated with the presence of an acridone fluorophore were analysed. Both fluorescence intensities are presented on a logarithmic scale.

In parallel to microscopic evaluation, flow cytometry analysis was performed to quantitate the effect of compounds on the membrane cell integrity and viability (Figure 5).

Our results indicate the increments of cellular fluorescence intensity of PI after *C. albicans* cells were treated with the octaarginine conjugate. The effect is time- (Figure 5) as well as concentration-dependent (data not shown) and correlated with an increase in the intensity of green fluorescence. Thus, one may conclude that the efficient accumulation of Compound **1-R8** resulted in fungal cell death in contrast to Compound **1** or **R8**. However, as indicated in Figure 5, there is a greater proportion of cells with an accumulated conjugate than PI-positive cells after 1 h and 3 h.

#### 2.3.3. Inhibition of the Relaxation Activity of Yeast Topoisomerase II In Vitro

Due to the known activity of acridine and acridone derivatives as human topoisomerase inhibitors widely analysed for cancer chemotherapy [14,17,31], we decided to analyse the influence of Compound **1** as well as its **R8** conjugate on the fungal equivalent of that enzyme. The relaxation of supercoiled plasmid DNA by yeast topoisomerase II (yTOPO II) was studied in the presence of different concentrations of both compounds.

**Figure 6:**
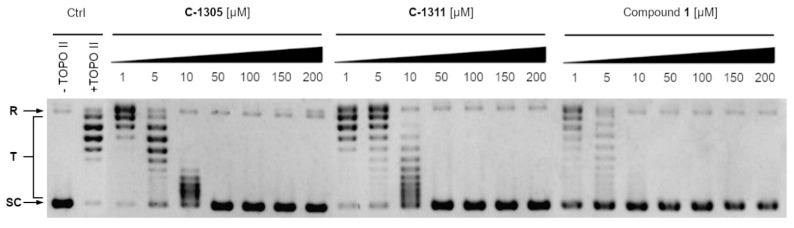
Inhibition of the catalytic activity of purified yeast DNA topoisomerase II by **C-1305**, **C-1311** and Compound **1** as measured by relaxation. Supercoiled pBR322 plasmid DNA (lane 1, -yTOPO II) was relaxed by purified yeast topoisomerase II in the absence (lane 2, +yTOPO II) or presence of analysed compounds at 1, 5, 10, 50, 100, 150 or 200 μM. The resulting topological forms of DNA were separated by gel electrophoresis. SC, supercoiled DNA; R, relaxed DNA; T, DNA topoisomers. Data shown are typical of three independent experiments.

The most effective Compound **1** totally inhibited the yeast topoisomerase II-mediated relaxation (Figure 6) at a concentration of 50 μM, lower than that detected for m-AMSA (IC_50_ > 200 μM) [15]. m-AMSA, an acridine derivative, was the first synthetic drug approved for clinical usage that was shown to act as a human topoisomerase II (hTOPO II) inhibitor [17]. The molecular mechanism of antitumor triazoloacridinone **C-1305** and imidazoacridinone **C-1311**, both acridone derivatives, also indicated its intercalation with DNA as well as the formula of a topo II-stabilizing complex [14,31]. It was probable that **C-1311** exhibited an inhibitory effect on yTOPO II (Figure 6), although its activity is not relevant for the antifungal activity due to the high MIC_90_ values (Table 1). Previously published data indicated **C-1311**’s inability to penetrate fungal cells to achieve the molecular target [8]. The lack of antifungal activity was also demonstrated for **C-1305**, despite its inhibitory effect on yTOPO II (Figure 6). **C-1311** as well as **C-1305** totally inhibited the yeast topoisomerase II-mediated relaxation (Figure 6) at a concentration of 50 μM. As previously reported, **C-1305** diminished the relaxation reaction by 50% at a concentration of 2.5 μM and totally inhibited human topoisomerase II-mediated relaxation at 10 μM [31]. The same level of hTOPO II inhibition ability was determined for **C-1311** (IC_50_ 2.5 μg mL^−1^; 6.5 μM) [14].

The previously reported modest inhibitory effect of m-AMSA on yTOPO II [15] indicates that the fungal enzyme might be sufficiently distinct from its human counterpart. Our results concerning yeast DNA topoisomerase II inhibition measured by relaxation for **C-1311** and **C-1305** also suggest differences in sensitivity to the presence of both compounds. Although the human enzyme seemed to be more sensitive than the yeast one, it is likely to find antifungal compounds that are inactive against the former but inhibit the latter. On the other hand, to enable selective targeting in order to become active, the antifungal topoisomerase inhibitor needs to enter into fungal cells to reach its intracellular targets. Our results indicate that the conjugation of Compound **1** with **R8** effectively increased its ability to enter fungal cells and made it possible to reach the molecular target, giving it a strong antifungal effect.

The molecular mechanism of antitumor triazoloacridinone **C-1305** and imidazoacridinone **C-1311** indicates the formula of a human topoisomerase II-stabilizing complex [14,31]. Cleavable, complex stabilizing drugs are the most important and valuable group of compounds used in the treatment of cancer. These agents, also called ‘poisons’, are able to stabilize a reversible, covalent DNA–topoisomerase II complex, which is a normal reaction intermediate in the catalytic cycle of the enzyme or are able to inhibit the religation step of the topoisomerase II catalytic cycle. We can distinguish drugs which form relatively short-lived complexes, such as etoposide, teniposide or m-AMSA, and compounds such as mitoxantrone or anthracyclines that form much longer-lived cleavable complexes with topoisomerase II [17].

**Figure 7:**
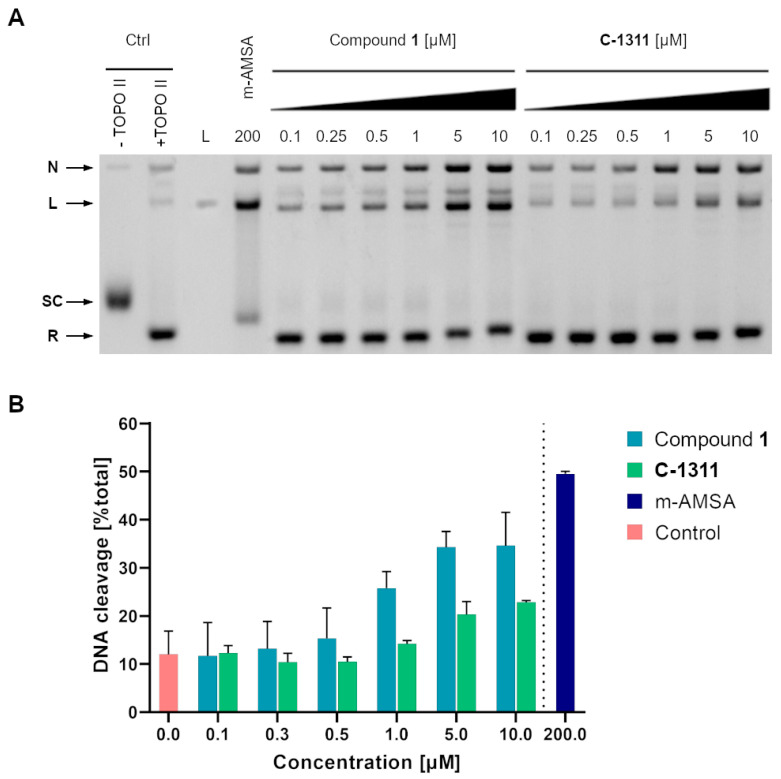
The influence of **C-1311** and Compound **1** on the formation of covalent DNA–yeast topoisomerase II complexes. (**A**) Supercoiled pBR322 plasmid DNA was incubated without (lane 1, ⁻ yTOPO II) or with purified yeast topoisomerase II in the absence (lane 2, +yTOPO II) or in the presence of 200 μM amsacrine (lane 4), as well as with 0.1, 0.25, 0.5, 1, 5, and 10 μM Compound **1** (lanes 5–10) or **C-1311** (lanes 11–16). The resulting DNA/yeast topoisomerase II complexes were digested by proteinase K, and the different topological forms of DNA were separated by agarose gel electrophoresis in the presence of ethidium bromide. Lane 3, linearized pBR322 DNA. S, supercoiled DNA; R, relaxed DNA; L, linear DNA; N, nicked circular DNA. Data shown are typical of two independent experiments. (**B**) Yeast topoisomerase II-mediated DNA cleavage in the presence of Compound **1**, **C-1311** and amsacrine. Plasmid DNA and purified yeast topoisomerase II were incubated with different concentrations of Compound **1**, **C-1311** and 200 μM amsacrine and the formation of linear DNA determined by gel densitometry. Data shown are the averages of two independent experiments. Control indicates the background levels of cleavable complexes formed in the absence of drug.

As indicated in Figure 7, Compound **1** as well as **C-1311** stimulated the formation of DNA–yeast topoisomerase II cleavable complexes in vitro.

The biologically active **C-1311** imidazoacridinone, as well as Compound **1,** is able to stabilize the formation of cleavable complexes between yeast topoisomerase II and pBR322 plasmid DNA (Figure 7A). Whatever the concentration, **C-1311** induced fewer cleavable complexes than Compound **1**. The cleavable complex formation was dose dependent (Figure 7B). Thus, our results indicate that Compound **1** may be considered as yeast topoisomerase II ‘poison’.

**Figure 8:**
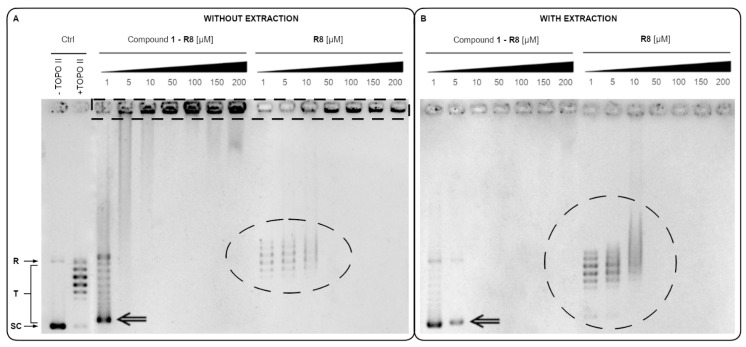
The effect of Compound **1-R8** and **R8** on yTOPO II relaxation ability. Results of parallel electrophoretic separation of samples from one experiment without the extraction step (**A**) and with the extraction step (**B**). Weak DNA–peptide interactions are highlighted with a dotted rectangle. Strong DNA–peptide interactions are highlighted with a dotted ellipse. Supercoiled pBR322 plasmid DNA migration delay is marked with arrows. SC, supercoiled DNA; R, relaxed DNA; T, DNA topoisomers. Data shown are typical of three independent experiments.

The inhibition of the catalytic activity of purified yeast DNA topoisomerase II by Compound **1-R8** conjugate as measured by relaxation seems not to be so obvious (Figure 8).

It was reported previously for hexaarginine peptide [42] and daunomycin-oligoarginine conjugate (Dau-Arg6) [43] that the electrostatic binding of ions is involved in the interaction between arginine residues and DNA. Positive charges of the Arg6 on the drug conjugate (Dau-Arg6) possess a natural tendency to be attracted by the negative DNA and extending contacts with this macromolecule. As indicated in Figure 8, DNA–peptide interactions are also important in the context of our studies. Electrophoresis of the relaxation assay samples without a previous extraction step (Figure 8, left panel) revealed that Compound **1-R8** and **R8** might interact with DNA due to the presence of positively charged octaarginine. Leaving protein impurities resulted in the concentration-dependent disappearance of the supercoiled pBR322 and the appearance of a strong signal in the gel wells (indicated by the dotted rectangle). Thus, we observe peptide–DNA interaction, which can be destroyed by the chloroform/isoamyl alcohol extraction step (no signal in the gel wells after extraction), but this is still not sufficient at higher concentrations (≥10 μM) to observe the presence of supercoiled pBR322 plasmid as well as its DNA topoisomers after relaxation by yTOPO II for Compound **1-R8**. As for **R8**, no plasmid was observed at any of the concentrations even after extraction. Moreover, for Compound **1-R8**, regardless of the deproteinization of the samples, a strong delay in gel mobility of supercoiled pBR322 plasmid was also detected (marked with arrows). A delay at a lower concentration of the Compound **1-R8** (1 μM) was observed for the samples without extraction, which suggests a significant influence of the DNA–peptide interaction on the relaxation process. A strong signal in the gel wells for the relaxation assay samples without a previous extraction step and disordered bands which cannot be removed by extraction (highlighted with an ellipse) were also detected for **R8** alone. Hypothetically, the presence of positively charged octaarginine, both conjugated and alone, resulted in compensation of DNA’s negative charge. In effect, the DNA–peptide complex was immobile during electrophoresis and remained in wells. Similar results were reported in studies on cell-penetrating peptides and DNA complexation [44].

### 2.4. Selectivity in Relation to Mammalian Cells

The investigated compounds were screened for their in vitro antiproliferative activity towards a human embryonic kidney cell line (HEK-293) and human liver cancer cell line (HEPG2) using colorimetric MTT. The results are presented in Table 5.

The obtained results show that **C-1311**, a known anticancer derivative, was highly active (IC_50_ ≤ 1 µM), while the rest of the compounds exhibited moderate activity (Compound **1** or Compound **1-R8**), or were, in general, inactive (**R8**) (IC_50_ > 50 µM) against both tested cell lines. The conjugation of Compound **1** with octaarginine (**R8**) resulted in at least a two-fold reduction in cytotoxicity with respect to Compound **1** itself (Table 5). Previously reported results indicated no cytotoxicity of the **R8** peptide on the HeLa cells [45]. This arginine-rich peptide seemed to be internalized in mammalian cells without causing serious perturbation to their membranes and viability. The same results were obtained by us for HEK-293, as well as HEPG2 cells.

To estimate the selectivity in relation to mammalian cells’ mycostatic selectivity index (MSI) was calculated as the ratio of IC_50_ to MIC_90_ values after converting the MIC value to micromolar concentrations. The results are presented in Table 6.

Positive mycostatic selectivity index values (MSI = IC_50_/MIC_90_) were obtained only for the Compound **1-R8** conjugate, ranging from 0.40 to 8.88. The highest MSI values were noted for *C. parapsilosis*.

As octaarginine (**R8**) is considered to be a membrane-active peptide, we have also decided to analyse Compound **1-R8** as well as **R8** hemolytic activity against human erythrocytes. Our results indicate that, opposite to Amphotericin B (EH_50_ 3.46 ± 0.15 μg mL^−1^), both compounds exhibited no hemolytic activity up to a concentration of 64 μg mL^−1^.

Summing up, the results clearly indicate that fungal cells can be effectively and selectively killed by our imidazoacridinone-based octaarginine conjugate.

## 3. Material and Methods

### 3.1. Chemical Synthesis

#### 3.1.1. General Formula for Imidazo- and Triazoloacridinone Synthesis

The products **C-1296**, **C-1305**, **C-1311**, **C-1410** and Compound **1** were obtained as the hydrochloride salt. Their structures were confirmed using spectral methods: mass spectrometry ESI-MS, nuclear magnetic resonance (^1^H and ^13^C NMR) and in some cases elemental analysis. The purity of these compounds was ascertained using thin-layer chromatography (TLC). Melting points were determined on a Stuart SMP30 capillary apparatus (SMP30—Stuart, Stone, UK). Mass spectra were recorded using an Agilent 6470A triple quadrupole (6470 Triple Quad LC/MS, Agilent Technologies, Waldbronn, Germany) LC/MS system (1260 Infinity II, Agilent Technologies, Waldbronn, Germany) with electrospray ionization source (ESI) in SCAN mode. Samples were prepared as 1 μg mL^−1^ solutions in methanol and were supplied in 1 μL aliquots to the mass spectrometer in the mixture of 38 % acetonitrile, 57 % water, 5 % formic acid (by volume) at a flow rate of 500 μL min^−1^. The ChemStation software was used to control the LC-MS system and for data processing (Agilent Technologies, Waldbronn, Germany).

^1^H and ^13^C NMR spectra were recorded on a Varian VXR-S spectrometer operating at 500 MHz (Varian Unity Inova 500, Palo Alto, CA, USA). Chemical shifts are reported as δ units in ppm downfield from internal tetramethylsilane. The NMR abbreviations used are as follows: br.s—broad signal, s—singlet, d—doublet, t—triplet, qu—quartet, qt—quintet, m—multiple. The results of the elemental analyses for individual elements fit within ±0.4% of theoretical values.

All aliphatic amines used in the synthesis of the described derivatives were purchased from Sigma Aldrich (Sigma Aldrich, St. Louise, MO, USA). Other reagents and solvents were obtained from POCH (POCH, Avantor, Gliwice, Poland).

##### Synthesis of 5-[-(3′-Dimethylamino)propylamino]-8-hydroxy-6H-[1,2,3]-triazolo[4,5,l-de]acridin-6-one (**C-1305**)

3-(Dimethylamino)-1-propylamine (1.93 mL, 15 mmol) was added to a suspension of 5-chloro-8-hydroxy-6H-[1,2,3]-triazolo[4,5,l-de]acridin-6-one (1.36 g, 5 mmol) in dry DMA (10 mL), and the mixture was heated with stirring at 60 °C for 2 h. Ethanol (20 mL) was added and the reaction mixture was left overnight in a refrigerator. The product was filtered off, and then was dissolved in methanol and prepared as dihydrochlorides by adding of mixture ether/HCl. Yield 73%; m.p. 228–230 °C; ^1^H NMR (Me_2_SO-d_6_) δ: 10.34 (s, 1 H, 8-OH), 9.32 (t, J = 6.35 Hz, 1H, 5-NH), 8.28–8.31 (m, 2H), 7.71 (s, 1H), 7.41 (dd, J_1_ = 8.8 Hz, J_2_ = 2.9 Hz, 1H), 7.16 (d, J = 8.3 Hz, 1H), 3.91 (qu, J = 6.8 Hz, 2H, NHCH_2_CH_2_), 3.16 (t, J = 7.8 Hz, 2H, CH_2_CH_2_N(CH_3_)_2_), 2.75 (s, 6H, CH_3_), 2.07–2.12 (m, 2H, NHCH_2_CH_2_CH_2_N(CH_3_)_2_). ^13^C NMR (DMSO-d_6_) δ: 177.26, 156.80, 152.72, 135.37, 131.29, 129.30, 128.27, 126.566, 122.93, 117.35, 112.20, 111.99, 99.40, 54.62, 42.53, 24.35. ESI-MS [M + H^+^] C_18_H_19_N_5_O_2_-337.9

##### Synthesis of 5-[-(3′-Dimethylamino)propylamino]-8-methyl-6H-[1,2,3]-triazolo[4,5,l-de]acridin-6-one (**C-1296**)

3-(Dimethylamino)-1-propylamine (0.96 mL, 7.5 mmol) was added to a suspension of 5-chloro-8-methyl-6H-[1,2,3]-triazolo[4,5,l-de]acridin-6-one (0.675 g, 2.5 mmol) in dry DMA (10 mL), and the mixture was heated with stirring at 60 °C for 3 h. Chloroform (75 mL) and water (40 mL) were added and the reaction mixture was vigorously shaken. The organic layer was separated, and water (75 mL) was added and acidified with L-lactic acid. After shaking, the water layer was separated, made basic with 1M NaOH, and extracted with chloroform. The organic layer was evaporated and was dissolved in methanol and prepared as dihydrochlorides by adding of mixture ether/HCl. Yield 62%; m.p. 120–122 °C; ^1^H NMR (Me_2_SO-d_6_) δ: 9.32 (br.s, 1H, 5-NH), 8.33 (d, J = 8.3 Hz, 1H), 8.25 (d, J = 9.1 Hz, 1H), 8.13 (s, 1H), 7.74 (dd, J_1_ = 8.2 Hz, J_2_ = 1.7 Hz, 1H), 7.13 (d, J = 9.3 Hz, 1H), 3.60–3.65 (m, 2H, NHCH_2_CH_2_), 3.17–3.21 (m, 2H, CH_2_CH_2_N(CH_3_)_2_), 2.79 (s, 6H, CH_3_), 2.48 (s, 3H, CH_3_-Ar) 2.12 (qt, J = 7.4 Hz, 2H, NHCH_2_CH_2_CH_2_N(CH_3_)_2_). The compound was poorly soluble in most solvents in amounts necessary for ^13^C NMR analysis. ^13^C NMR (D_2_O + TFA) δ: 177.45, 153.784, 139.50, 136.93, 135.57, 132.52, 131.28, 129.95, 127.87, 124.35, 117.65, 114.24, 99.29, 57.10, 22.95, 21.82, 17.69. ESI-MS [M+H^+^] C_19_H_21_N_5_O–336.2

##### Synthesis of 5-[2′-(Ethyl)-ethylenediamine]-8-hydroxy-6H-[1,2,3]-triazolo[4,5,l-de]acridin-6-one (**C-1410**)

N-Ethylethylenediamine (1.57 mL, 15 mmol) was added to a suspension of 5-chloro-8-hydroxy-6H-[1,2,3]-triazolo[4,5,l-de]acridin-6-one (1.36 g, 5 mmol) in dry DMSO (15 mL), and the mixture was heated with stirring at 70 °C for 2 h. After cooling, water (20 mL) was added, and the reaction mixture was extracted with chloroform. The chloroform layer was acidified with L-lactic acid and extracted with water. The water layer was made basic with aqueous NaOH and extracted with chloroform. The organic layer was evaporated and dissolved in methanol and prepared as hydrochlorides by adding a mixture of ether/HCl. Yield 90%; m.p. 300 °C; ^1^H NMR (Me_2_SO-d_6_) δ: 9.35–9.40 (br.s, 1H, 5-NH), 8.34–8.36 (m, 2H), 7.75 (s, 1H), 7.44 (dd, J_1_ = 8.9 Hz, J_2_ = 3.1 Hz, 1H), 7.25 (d, J = 9.3 Hz, 1H), 3.82 (qu, J = 7.4 Hz, 2H, NHCH_2_CH_2_NH(CH_2_CH_3_)), 3.17–3.21 (qt, J = 6.2 Hz, 2H, NHCH_2_CH_2_NH(CH_2_CH_3_)), 3.00–3.04 (m, 2H, NHCH_2_CH_2_NH(CH_2_CH_3_)), 1.20 (t, J = 7.3 Hz, 3H, NHCH_2_CH_2_NH(CH_2_CH_3_)). ^13^C NMR (DMSO-d_6_, Temp. 80.0 °C) δ: 177.58, 156.94, 152.70, 135.92, 131.46, 129.24, 128.54, 126.65, 123.12, 117.29, 112.33, 112.13, 100.17, 45.78, 42.85, 11.32. ESI-MS [M+H^+^] C_17_H_17_N_5_O_2_–324.1; elemental analysis: C_17_H_19_N_5_O_2_Cl_2_

##### Synthesis of 5-((2-(Diethylamino)ethyl)amino)-8-hydroxy-6H-imidazo[4,5,1-de]acridin-6-one (**C-1311**)

N,N-Diethylethylenediamine (1.43 mL, 10 mmol) was added to a suspension of 1-chloro-7-hydroxy-4-nitro-9 (10H)-acridone (0.73 g, 2.5 mmol) in 10 mL of DMSO, the solution was stirred at 60–80 °C for 0.5 h, and then 40% MeOH solution (50 mL) was added and stirred again at room temperature for 0.5 h. The reaction mixture was filtered, washed with cold methanol and allowed to dry. The derivative obtained earlier (0.813 g, 2 mmol), 10% Pd/C (0.8 g), and 30 mL of 96% formic acid were hydrogenated by bubbling hydrogen gas at room temperature for 24 h. After this time, the catalyst was filtered off, and about 1–2 mL of concentrated HCl was added to the filtrate and heated at 110 °C for 24 h. Formic acid was evaporated, and the obtained residue was heated for 3 h in a 1/1 mixture of water and methanol (about 50 mL). The solvents were evaporated. Methanol was added to the residue and acidified with concentrated hydrochloric acid. The product was crystallized from acetone. Yield 68%; m.p. 278–280 °C; ^1^H NMR (Me_2_SO-d_6_) δ: 10.69 (s, 1H, 8-OH), 9.54 (s, 1H, N10CH), 8.99 (s, 1H, 5-NH), 8.31 (d, J = 8.8 Hz, 1H), 8.05 (d, J = 9.1 Hz, 1H), 7.75 (s, 1H), 7.40 (dd, J_1_ = 8.8 Hz, J_2_ = 2.5 Hz, 1H), 7.12 (d, J = 9.1 Hz, 1H), 3.90–3.96 (m, 2H, NHCH_2_CH_2_N(CH_2_CH_3_)_2_), 3.30–3.34 (m, 2H, NHCH_2_CH_2_N(CH_2_CH_3_)_2_), 3.17–3.26 (m, 4H, NHCH_2_CH_2_N(CH_2_CH_3_)_2_), 1.25 (t, J = 7.1 Hz, 6H, NHCH_2_CH_2_N(CH_2_CH_3_)_2_). ^13^C NMR (MeOH-d_6_) δ: 173.84, 157.96, 150.44, 133.04, 125.66, 123.62, 122.08, 119.79, 118.13, 116.22, 113.94, 111.91, 111.67, 100.05, 50.18, 37.66, 12.22. ESI-MS [M+H^+^] C_20_H_22_N_4_O_2_–350.9

##### Synthesis of 5-((2-Aminoethyl)amino)-8-hydroxy-6H-imidazo[4,5,1-de]acridin-6-one (Compound **1**)

N,N-Ethylenediamine (0.89 mL, 13.34 mmol) was added to a suspension of 1-chloro-7-hydroxy-4-nitro-9 (10H)-acridone (1 g, 3.44 mmol) in 15 mL of DMSO, the solution was stirred at room temperature for 2.5 h, and then 40% MeOH solution (50 mL) was added and stirred again at room temperature for 0.5 h. After this time, water was added (~50 mL) and the reaction mixture was stirred for 0.5 h. The precipitate was collected by filtration. Next, it was transferred into water, acidified with a dilute hydrochloric acid, and stirred for 0.5 h. Undissolved material was filtered off, and the solution was evaporated to a small volume. The product was precipitated out using acetone (~100 mL), and then was filtered off to give 0.72 g (68%). The derivative obtained earlier (0.72 g. 2.3 mmol), 10% Pd/C (0.7 g), and 50 mL of 96% formic acid were hydrogenated by bubbling hydrogen gas at room temperature for 24 h. After this time, the catalyst was filtered off, and about 1–2 mL of concentrated HCl was added to the filtrate and heated at 110 °C for 24 h. Formic acid was evaporated, and the obtained residue was heated for 3 h in a 1/1 mixture of water and methanol (about 50 mL). The solvents were evaporated. Methanol was added to the residue and acidified with concentrated hydrochloric acid. The product was crystallized from diethyl ether. Yield 59%; m.p. 332–334 °C; ^1^H NMR (Me_2_SO-d_6_) δ: 10.27 (s, 1H, N10CH), 9.19 (br.s, 1H, 5-NH), 8.34 (d, J = 9.3 Hz, 1H), 8.10 (d, J = 8.8 Hz, 1H), 7.98 (br.s, 3H, NHCH_2_CH_2_NH_3_^+^), 7.77 (s, 1H), 7.41 (dd, J_1_ = 8.8 Hz, J_2_ = 2.9 Hz, 1H), 7.23 (d, J = 9.3 Hz, 1H), 3.78 (t, J = 6.1 Hz, 2H, NHCH_2_CH_2_NH_2_), 3.11 (t, J = 5.9 Hz, 2H, NHCH_2_CH_2_NH_2_). ^13^C NMR (D_2_O + TFA) δ: 175.41, 155.52, 150.06, 130.73, 127.74, 125.20, 124.22, 123.94, 121.70, 117.84, 116.88, 111.95, 110.34, 99.33, 39.37, 37.50 ESI-MS [M+H^+^] C_16_H_14_N_4_O_2_–295.0

#### 3.1.2. **R8**-Conjugated Compound **1** Synthesis and Purification

Compound **1** was derivatized with Fmoc. Compound **1** (1 eq), Fmoc-Cl (1.5 eq; Sigma Aldrich, St. Louis, MO, USA) and DIPEA (3 eq, N,N-diisopropylethylamine, Iris Biotech GmbH, Marktredwitz, Germany) were dissolved in DMF (N,N-dimethylformamide, POCH, Avantor, Gliwice, Poland). The reaction was carried out for 1 h at RT and completeness was confirmed with LC-MS. Fmoc-derivatized Compound **1** was attached to 2-CTC resin (loading 0.73 mmol g^−1^, Sunresin, Sunresin Park, Xi’an Hi-tech Industrial Development Zone, Shaanxi, China). Crude mixture was diluted four-fold in DMF and added to 2-CTC resin (0.1 mmol of Compound **1** derivative per 1 g of the resin). Resin was agitated for 12 h at RT. Unreacted resin was end-capped with methanol (Chempur, Piekary Slaskie, Poland) and DIPEA in DCM (dichloromethane, Chempur, Piekary Slaskie, Poland; 2:1:17 by volume, approx. 10 mL of the mixture per gram of resin) with agitation for 30 min at RT. Subsequently, resin was filtered with three volumes of DMF and DCM. The estimated substitution of the resin was 0.05 mmol g^−1^. Deprotection of the Fmoc group was accomplished with a 20% piperidine (Iris Biotech GmbH, Marktredwitz, Germany) solution in DMF for 15 min. The amino acid used in the study was Fmoc-L-Arg(Pbf)-OH (Orpegen Peptide Chemicals GmbH, Heidelberg, Germany). The conjugate was synthesized on 2-chlorotritile chloride resin. Each acylation was performed twice with a equimolar mixture of DIC (N,N’-diisopropylcarbodiimide, Carbolution Chemicals GmbH, St. Ingbert, Germany), OxymaPure (Carbolution Chemicals GmbH, St. Ingbert, Germany), and Fmoc-L-Arg(Pbf)-OH dissolved in DCM:DMF (1:1, by volume) in four-fold excess based on the resin for 1.5 h. After deprotection and coupling reactions, the resin was rinsed with DMF and DCM, and subsequently the chloranil test was carried out. The conjugate was cleaved from the resin using a mixture of TFA (Apollo Scientific, Denton, UK), TIS (Iris Biotech GmbH, Marktredwitz, Germany) and demineralized water (95:2.5:2.5, by volume). This stage was accomplished at RT within 1.5 h with agitation. Crude conjugate was precipitated with cooled diethyl ether (POCH, Avantor, Gliwice, Poland) and centrifuged. The precipitate was dissolved in water and lyophilized. The reaction scheme for the synthesis of Compound **1-R8** is presented in the Supplementary Materials in Figure S3. Purification was performed by RP-HPLC (C18 column, gradient grade water and acetonitrile with 0.1% TFA, by volume). Eluents were purchased from POCH (Avantor, Gliwice, Poland). Identity was confirmed with ESI-MS (Waters Alliance e2695 system with Acquity QDA detector, Waters, Milford, MA, USA; positive ion mode; 50–1250 *m*/*z* range). Pure fractions were collected (>95%, by RP-HPLC) and lyophilized (Table S1).

The purity of the conjugate was determined on a Varian ProStar HPLC system (SpectraLab Scientific Inc., Markham, ON, Canada) controlled by a Galaxie Chromatography Data System. Analyses were carried out on a Phenomenex Luna C18(2) column (4.6 × 150 mm, 110 Å pore size, 5 μm particle size). The solvent systems used were: 0.1% aqueous TFA (A) and 0.1% TFA in acetonitrile (B). The peptides were eluted with a linear gradient 10–100% B in A over 10 min at 25 ± 0.1 °C. UV detection at 214 nm was used and the mobile phase flow rate was 2.0 mL min^−1^ (Figure S4).

To additionally confirm that the arginine residue is conjugated with the primary amino group of AEA moiety, ^1^H NMR and COSY analysis were performed for the Compound **1-R** conjugate (Supplementary Materials). The structure was determined with the use of Bruker AVANCE III at 500 MHz (Bruker Polska Sp. z.o.o., Poznan, Poland) and DMSO-d_6_ was used as the solvent. ^1^H NMR (500 MHz, DMSO-d_6_): 1.49 (2H, m, J = 6.6 Hz, J = 12.6 Hz, H_γ_); 1.72 (2H, s, H_β_); 3.10 (2H, q, J = 6.6 Hz, J = 12.6 Hz, H_δ_); 3.44 (1H, m, CH_b_-AEA); 3.51 (1H, m, CH_a_-AEA); 3.58 (2H, m, CH_2_-AEA); 3.75 (1H, m, H_α_); 6.97 (1H, d, J = 8.9 Hz, aromatic); 7.38 (1H, dd, J = 2.8 Hz, J = 8.8 Hz, aromatic); 7.58 (1H, t, NH, J = 5.8 Hz–guanidinium); 7.73 (1H, d, J = 2.8 Hz aromatic); 8.02 (1H, d, J = 8.9 Hz, aromatic); 8.17 (2H, m, NH_2α_); 8.30 (1H, d, J = 8.9 Hz, aromatic); 8.77 (1H, t, J = 5.7 Hz, NH-amide); 8.99 (1H, t, J = 5.3 Hz NH-AEA); 9.16 (1H, s, aromatic).

### 3.2. Microorganisms Strains and Growth Conditions

The following fungal strains were used: *C. albicans* ATCC 10231, *C. glabrata* ATCC 90030, *C. krusei* ATCC 6258, *C. parapsilosis* ATCC 22019, *S. cerevisiae* ATCC 9763. Fungal strains used in this investigation were routinely grown over 18 h at 30 °C in YPG liquid medium (1% yeast extract, 1% peptone, 2% glucose) in a shaking incubator (INFORS HT Bottmingen, Switzerland). For growth on solid media, 1.5% agar was added to the YPG medium.

### 3.3. Antimicrobial Activity Assay

Antifungal in vitro activity was determined by the modified M27-A3 specified by the CLSI [46]. Wells containing serially diluted examined compounds and compound-free controls were inoculated with 12 h cultures of tested strains to the final concentration of 10^4^ fungi colony-forming units (CFU) mL^−1^. Plates were incubated for 24 h at 37 °C and growth was then quantified by measuring the optical density at 600 nm, using a microplate reader (TECAN Spark 10M, Grödig, Austria). The MIC was defined as the lowest drug concentration at which at least a 90% decrease in turbidity, in comparison to the drug-free control, was observed. Antifungal activity was determined in RPMI-1640 (Sigma-Aldrich, St. Louis, MO, USA) medium buffered to pH 7.0. The final concentration of the compound solvent (DMSO) did not exceed 2.5% volume of final suspension in each well and did not influence the growth of microorganism.

### 3.4. Fungicidal Activity Analysis

Minimum fungicidal concentrations (MFCs) were determined as described previously [7] by spot assay. The MFC was determined as the lowest concentration of the test compound in which no recovery of microorganisms was observed.

Killing kinetics analysis was performed as described previously [7]. Briefly, the suspensions of *Candida albicans* ATCC 10231 cells (500 μL) in RPMI 1640 at cell density of 10^4^ cells mL^−1^ were added to 500 μL of RPMI 1640 medium with various concentrations of the compounds, corresponding to 8 × MIC, 4 × MIC, 2 × MIC, MIC, 1/2 × MIC, vigorously shaken, and then incubated at 37 °C for 60, 180 and 300 min. After an appropriate time of incubation, 10 μL was withdrawn and spread on YPG agar plates. The number of colony-forming units (CFUs) was determined after overnight incubation of the plates at 37 °C. The killing kinetics analysis was performed with the GraphPad Prism 8 software (GraphPad Software Inc., San Diego, CA, USA).

### 3.5. Monitoring of the Cellular Uptake

*Candida albicans* ATCC 10231 strain were grown in the YPG medium overnight (16–18 h) at 30 °C, washed twice with sterile phosphate-buffered saline (PBS) and resuspended in RPMI 1640 at a cell density of 2 × 10^7^ cells mL^−1^. The inoculum (5 mL) was added to 5 mL RPMI 1640 with 256 μg mL^−1^ of tested compounds. After appropriate period of time, 2 mL of cell suspension was washed three times with sterile phosphate-buffered saline (PBS). If appropriate, cells where resuspended in 1 mL of PBS and 5 μL of 2 mg mL^−1^ propidium iodide (PI, Sigma-Aldrich, St. Louis, MO, USA) were added and incubated for 30 min and then rinsed three times with PBS buffer. After that, cells were resuspended in 5 μL 90% (by volume) glycerol/10% (by volume) 1 × PBS and transferred to a microscopic slide. The cells were examined with the Olympus BX-60 fluorescence microscope (λ_ex_ = 330–385 nm, λ_em_ ≥ 420 nm, lens × 40) equipped with the Olympus XC50 digital camera (Olympus Corporation, Tokyo, Japan) or LSM800 T-PMT confocal microscope with a CCD camera (ZEISS, Göttingen, Germany). Images were analysed and processed with cellSens Dimension and ZEN Blue software [47].

### 3.6. Flow Cytometry Analysis

The change in fungal membrane permeability was determined by propidium iodide influx assay. *Candida albicans* ATCC 10231 strain (10^6^ cells mL^−1^) in RPMI 1640 medium was incubated with 10 μM of Compound **1-R8** or 50 μM of Compound **1** for 0, 1 h and 3 h. Cells were harvested by centrifugation (2300× *g*, 5 min) and washed twice with PBS buffer. Then, cells were resuspended in 1 mL of PBS with 1 μL mL^−1^ propidium iodide (final concentration) and incubated for 30 min. A total of 300 μL of cell suspension were analysed using a Guava easyCyte 8 flow cytometer (Merck Millipore, Hayward, CA, USA). Data were processed with FlowJo™ v10.8.0 Software (BD Life Sciences, Franklin Lakes, NJ, USA) [48].

### 3.7. Yeast Topoisomerase II Relaxation Assay and Inhibition

The inhibition of yeast topoisomerase II was analysed according to the relaxation assay kit from Inspiralis (Inspiralis Limited, Norwich, UK). Briefly, 250 ng of supercoiled pBR322 DNA (Thermo Fisher Scientific, Waltham, MA, USA), 1 mM ATP (Inspiralis Limited, UK), and 1–200 μM of the analysed compound were mixed with reaction buffer (1 mM Tris-HCl (pH 7.9), 10 mM KCl, 0.5 mM MgCl_2_, 0.2 % (by volume) glycerol). The reaction was initiated by the addition of an enzyme, allowed to proceed at 30 °C for 30 min and terminated by addition of 40% (*m*/*V*) sucrose, 100 mM Tris-HCl pH 8, 10 mM EDTA, and 0.5 mg mL^−1^ Bromophenol Blue. Two-step extraction with chloroform:isoamyl alcohol (24:1, by volume) and butanol water was performed and mixtures were analysed on 1% agarose gel in 1 × TAE buffer, 3h, 4.5 V cm^−1^. Gel was stained in GelRed 3 × staining solution (Biotium, Fremont, CA, USA) for 30 min and photographed with Gel Doc XR+Gel Documentation System (Bio-Rad: Hercules, CA, USA).

### 3.8. Formation of Cleavable Complexes In Vitro

Cleavage assay of yeast topoisomerase II was performed according to the formation of cleavable complexes kit from Inspiralis. The experimental conditions were the same as for the relaxation assay except for the amount of yeast topoisomerase II (16 times more enzyme was used). The reaction was initiated by the addition of the enzyme and allowed to proceed for 10 min at 30 °C. Then, 0.35% SDS and 0.3 mg mL^−1^ proteinase K (A&A Biotechnology, Gdynia, Poland) was added, and the probes were incubated in 56 °C for 1 h. The reaction was terminated by the addition of 100 mM Tris-HCl pH 8, 10 mM EDTA, 0.5 mg mL^−1^ Bromophenol Blue. Extraction with chloroform:isoamyl alcohol (24:1, by volume) was performed, and mixtures were analysed on the 1.2% agarose gel with 0.5 µg mL^−1^ ethidium bromide in 1x TAE buffer, 18 h, 1.1 V cm^−1^. Gel was photographed with the Gel Doc XR+Gel Documentation System.

### 3.9. Antiproliferative Activity Determination

The human embryonic kidney cell line (HEK-293) and human liver cancer cell line (HEPG2) were purchased from ATCC (Manassas, Virginia, USA). HEK-293 was cultured in Dulbecco’s Modified Eagle Medium (Corning, NY, USA) and HEPG2 in Minimum Essential Medium Eagle (Corning, NY, USA). For each cell line, the culture medium was supplemented with 10% fetal bovine serum, 2 mM L-glutamine, and antibiotics: penicillin 62.6 µg mL^−1^ and streptomycin 40 µg mL^−1^. The cells were cultured at the humidified atmosphere of 5% CO_2_/95% air and routinely tested for *Mycoplasma* contamination.

Antiproliferative activity of the compounds was determined by (3-(4,5-dimethylthiazol-2-yl)-2,5-diphenyltetrazolium bromide (MTT, Sigma-Aldrich, St. Louis, MO, USA) assay, which involves the assessment of cell viability by the measurement of the cellular oxidoreductase activity. Briefly, cells were seeded into 96-well culture plates and were allowed to adhere overnight. The investigated compounds were dissolved in DMSO and added to wells (1% final concentration of DMSO) in various concentrations. After 72 h incubation, 20 µL of MTT solution in PBS (4 mg mL^−1^) was added to each well, and plates were incubated for an additional 3h in standard culture conditions. After that, the medium was aspirated, and formazan crystals were dissolved in DMSO. The absorbance was determined at 570 nm with a microplate reader (Asys UVM 340 Microplate Reader, Biochrom, Cambridge, UK). All experiments were performed three times independently in triplicate.

### 3.10. Hemolytic Activity Determination

Red blood cell concentrates were kindly provided by the Regional Centre for Blood Donation and Blood Treatment in Gdansk. The haemolytic activity determination was carried out by the serial dilution method according to the procedure described earlier [49]. Human erythrocytes were suspended in a solution of saline to obtain a cell density of suspension 2 × l0^7^ per mL. Suitable amounts of solutions of compounds diluted in DMSO were added to the cell suspension in tubes, incubated at 37 °C for 30 min, and then centrifuged (1700× *g*, 5 min, 4 °C). The concentration of haemoglobin in the supernatant after centrifugation of the erythrocytes suspension was determined by measuring the absorbance at wavelength λ = 540 nm (A_540_ ^sample^). Control experiments with the same amount of DMSO were also performed (A_540_ ^DMSO^). The maximum level of haemolysis was obtained after incubation of the cell suspension in the presence of 0.1% Triton X-100 (positive control, A_540_ ^0.1%Triton X−100^). Normal saline (0.9%) was used as the negative control (0% lysis, A_540_ ^0.9% NaCl^). The EH_50_ values for each compound were calculated with GraphPad Prism 8 software. The EH_50_ is the interpolated concentration of compound, for which the A_540_ value is exactly 50% of the A_540_ value measured for the positive control sample. EH (%) was calculated as follows:

EH (%) = ((A_540_ ^sample^ − A_540_ ^DMSO^)/(A_540_ ^0.1%Triton X−100^ − A_540_ ^0.9%NaCl^)) × 100

## 4. Conclusions

Conjugation of Compound **1** with octaarginine markedly changed the biological properties of this imidazoacridinone derivative. Particularly, unlike the mother molecule, its conjugate with the cell-penetrating peptide (CPP) exhibited strong antifungal activity, also for fluconazole-resistant strains. The effect seems to be unrelated only to simple cytoplasmic membrane damage by used CPP (**R8**) or Compound **1-R8** since no cytotoxicity of the **R8** peptide on mammalian cell lines was reported previously [45] or presented in this report, as well as there being no haemolytic activity for both compounds detected by us. On the other hand, the antifungal growth inhibitory effect of the conjugate Compound **1-R8** was clearly related to its facilitated uptake by sensitive fungal cells. Moreover, positive mycostatic selectivity index values determined by us clearly indicate that fungal cells can be effectively and selectively killed by our imidazoacridinone-based octaarginine conjugate.

Due to the fact that Compound **1** is a close derivative of **C-1311**, known human topoisomerase II inhibitor [14], we suppose that Compound **1-R8** antifungal activity may be related to its inhibitory effect on the fungal equivalent of that enzyme. Compound **1** was found to be a more effective yeast type II DNA topoisomerase inhibitor than **C-1311** and **C-1305**. It should be noted, however, that the interaction with this probable molecular target requires intracellular Compound **1** release from its conjugate with **R8**. The conjugation may facilitate the interaction with fungal topoisomerase II, while happily, possible release in mammalian cells is not a prerequisite for effective antiproliferative activity.

Summing up, we were able to show proof of concept that conjugation of CPP to acridine derivatives may result in the discovery of a new group of selective antifungal drugs, despite the use of controversial acridine derivatives, generally recognized as cytotoxic. Moreover, the clinical strains of *C. albicans* resistant to FLU, due to the FLU-induced overexpression of Cdr1p/Cdr2p drug efflux pumps as well as Mdr1p membrane transport protein of the major facilitator superfamily, remained sensitive to our novel compound. This finding suggests that the conjugate is not a good substrate for Cdr1p/Cdr2p and Mdr1p.

Future investigations will be focused on understanding the fundamental molecular mechanism of action of the tested conjugate and the synthesis of a set of similar compounds to understand the uniqueness of antifungal activity.

## Figures and Tables

**Table 1:** Antifungal activity of **C-1305** and **C-1311** and their derivatives against reference strains. MIC_90_, minimal inhibitory concentrations at which 90% of cells growth were inhibited. * > Means no activity at the concentration mentioned. The experiments were performed with at least five replicates.

Compound	* MIC_90_ (μg mL^−1^)				
	*Candida albicans* ATCC 10231	*Candida glabrata* ATCC 90030	*Candida krusei* ATCC 6258	*Candida parapsilosis* ATCC 22019	*Saccharomyces cerevisiae* ATCC 9763
**C-1305**	>64	>64	>64	>64	>64
**C-1311**	>64	>64	>64	>64	>64
**C-1296**	>64	>64	>64	>64	32
**C-1410**	>64	>64	>64	>64	>64
Compound **1**	>64	>64	>64	>64	>64
Compound **1-R8**	1	4	0.5	0.25	1
**R8**	>64	>64	>64	64	64
Amphotericin B	0.5	1	1	1	0.5

**Table 2:** Antifungal activity of Compound **1** and its octaarginine conjugate Compound **1-R8** against clinical strains in comparison with *C. albicans* ATCC 10231. * > Means no activity at the concentration mentioned. The experiments were performed with at least five replicates.

Compound	* MIC_90_ (μg mL^−1^)						
	*Candida albicans* ATCC 10231	*Candida albicans* B3	*Candida albicans* B4	*Candida albicans* Gu4	*Candida albicans* Gu5	*Candida albicans* F2	*Candida albicans* F5
Compound **1**	>64	>64	>64	>64	>64	>64	>64
Compound **1-R8**	0.5	2	2	2	2	1	1
**R8**	>64	>64	>64	>64	>64	>64	>64
Fluconazole	8	1	16	4	>64	8	>64

**Table 3:** The level of antifungal activity of analysed compounds and their consensus LogP _o/w_ indexes. * Average LogP of the five predictions [38]. ** (−) Means no antifungal activity; (±) low activity; (+) moderate activity; (++) high activity at the concentration mentioned. *** The structures and activity data published previously [8].

Compound	Consensus * LogP _o/w_	Antifungal Activity **
Compound **1**	0.52	−
**C-1410**	1.59	−
**C-1305**	1.72	−
**C-1311**	1.84	−
**C-1296**	2.47	±
**C-1330** ***	3.04	+
**C-1415** ***	3.06	+
**C-1558** ***	4.25	+
Compound **1-R8**	−5.96	++

**Table 4:** Fungicidal activity of Compound **1-R8** and amphotericin B (MFC - minimal fungicidal concentration that kills 99% of cells). * > Means no 99% killing activity at the concentration mentioned. The experiments were performed at least in three replicates.

Compound	* MFC (μg mL^−1^)				
	*C. albicans* ATCC 10231	*C. glabrata* DSM 11226	*C. krusei* DSM 6128	*C. parapsilosis* DSM 5784	*S. cerevisiae* ATCC 9763
Compound **1-R8**	32	128	16	8	2
**Amphotericin B**	2	2	2	2	2

**Table 5:** In vitro antiproliferative activity (IC_50_ ± SD (μM)) of selected compounds toward human embryonic kidney cells (HEK-293) and human liver cancer cells (HEPG2) after 72 h treatment. The experiments were performed with three replicates. * IC_50_—the half-maximal inhibitory concentration.

Compound	* IC_50_ μM	
	HEK293	HEPG2
Compound **1**	0.550 ± 0.125	0.364 ± 0.013
Compound **1-R8**	1.390 ± 0.099	0.991 ± 0.097
**R8**	>50	>50
**C-1311**	0.008 ± 0.007	0.129 ± 0.037

**Table 6:** Mycostatic selectivity in relation to mammalian cell lines.

Compound	IC_50_ HEK293/MIC_90_				
	*Candida albicans* ATCC 10231	*Candida glabrata* ATCC 90030	*Candida krusei* ATCC 6258	*Candida parapsilosis* ATCC 22019	*Saccharomyces cerevisiae* ATCC 9763
**R8**	-	-	-	-	-
**C-1311**	<0.00005	<0.00005	<0.00005	<0.00005	<0.00005
Compound **1**	<0.0058	<0.0058	<0.0058	<0.0058	<0.0058
Compound **1-R8**	2.22	0.56	4.44	8.88	2.22
	IC_50_ HEPG2/MIC_90_				
**R8**	-	-	-	-	-
**C-1311**	<0.00078	<0.00078	<0.00078	<0.00078	<0.00078
Compound **1**	<0.0038	<0.0038	<0.0038	<0.0038	<0.0038
Compound **1-R8**	1.58	0.40	3.17	6.33	1.58

## Data Availability

All data generated or analysed during this study are available from the corresponding author by request.

## Supplementary Materials

The following are available online at https://www.mdpi.com/article/10.3390/ijms222413190/s1.
