# Supplementary material for: The Effect of Conjugation with Octaarginine, a Cell-Penetrating Peptide on Antifungal Activity of Imidazoacridinone Derivative
Source: Int J Mol Sci. 2021 Dec 7;22(24):13190. doi: 10.3390/ijms222413190 (PMC8705783; doi:10.3390/ijms222413190)
Supplement: Supplementary file 1 [file ijms-22-13190-s001.zip › ijms-1469307-supplementary.pdf]

Supplementary data

The effect of conjugation with octaarginine, a cell-penetrating peptide on antifungal activity of imidazoacridinone derivative

Kamila Rząd[1], Ewa Paluszkiewicz[1], Damian Neubauer[2], Mateusz Olszewski[1], Katarzyna Kozłowska-Tylingo[1], Wojciech Kamysz[2], Iwona Gabriel[1],*

[1]Department of Pharmaceutical Technology and Biochemistry, Faculty of Chemistry and BioTechMed Center, Gdańsk University of Technology, 11/12 Narutowicza Str. , 80-233 Gdańsk, Poland

[2]Department of Inorganic Chemistry, Medical University of Gdańsk, Faculty of Pharmacy, Gen. J. Hallera 107th Avenue , 80-416, Gdańsk, Poland

Table of contents:

1. **The synthesis of C-1296, C-1305, C-1311, C-1410 and Compound 1…………………………….....2**
   1.1. **The synthesis of triazoloacridinone derivatives…………………………………………………….2**
   1.2. **The synthesis of imidazoacridinone derivatives……………………………….…….………...2**
   1.3. **^{1}H NMR, ^{13}C NMR and ESI-MS Spectra for imidazo and triazoloacridinone derivatives…………………………………………………………………………….…….3**
2. **Compound 1-R8 synthesis and chemical analysis…………………………………….……………11**
3. **Compound1-R ^{1}H NMR and COSY analysis…………………………………………………………12**

1. **The synthesis of C-1296, C-1305, C-1311, C-1410 and Compound 1**
   1.1. **The synthesis of triazoloacridinone derivatives**

The 8-substituted *5*-[(aminoalkyl)amino]-6H-v- triazolo [4,5,l-de]-acridin-6-ones (C-1305, C-1296 and C-1410) presented in Scheme I were prepared according to the previously reported procedure [Cholody, W.M., Martelli, S., Konopa, J., 8-Substituted 5[(aminoalkyl-amino-6H-[1,2,3]-triazolo[4,5,l-de]acridin-6-ones as potential antineoplastic agents. Synthesis and biological activity. J. Med. Chem., 1990, 33, 2852-2856, doi:10.1021/jm00172a028]. The required starting materials 1-chloro-4-nitro-9(10H)-acridinones **(I)** was prepared on the basis of the method published earlier [Capps D. B., Dunbar J., Kesten S. R., Shillis J., Werbel L. M., 2-(Aminoalkyl)-5-nitropyrazolo[3,4,5-kl]-acridines, a New Class of Anticancer Agents, J. Med. Chem., 1992, *35*, 4770-4778]. The studied derivatives of 5-amino-6H-v-triazolo[4,5,l-de]acridin-6-one were prepared by the condensation of triazoloacridinones (**III**) with suitable aliphatic amines in DMSO or DMA. The compounds were obtained as hydrochlorides.

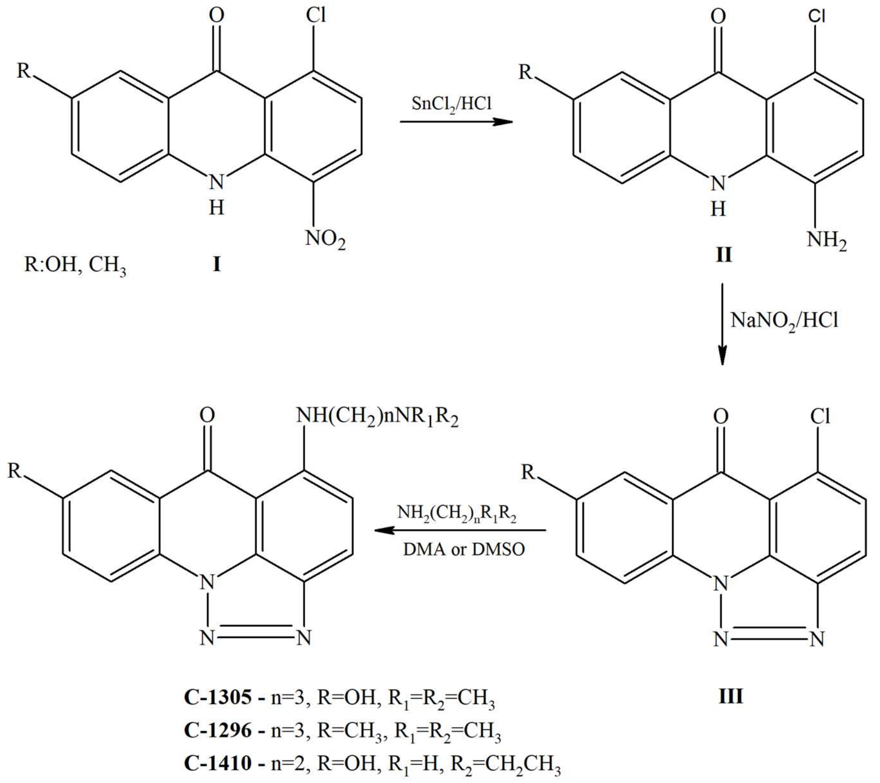

Figure S1. Strategy of the synthesis of compounds **C-1305**, **C-1296** and **C-1410.**

   1.2. **The synthesis of imidazoacridinone derivatives**

Compounds **C-1311** and **Compound 1** were synthesized starting from 1-chloro-7-hydroxy-4-nitro-9(10H)-acridinone, through the substitution of aliphatic amines, reduction and cyclization of the resulting derivatives. This method of synthesis was described previously [Chołody W.M., Martelli S., Pardziej-Lukowicz J. Konopa J., 5-[(Aminoalkyl)amino]imidazo[4,5,1-de]acridin-6-ones as a novel class of antineoplastic agents. Synthesis and biological activity, J. Med. Chem., 1990, 33, 49-52, doi:10.1021/jm00163a009; Konieczny M.T., Konopa J.K., Acridone derivatives and preparation of 8-hydroxy-imidazoacridinone derivatives, 1998, GB 2317888]. The strategy of **C-1311** and **Compound 1** synthesis is presented on Figure S2.

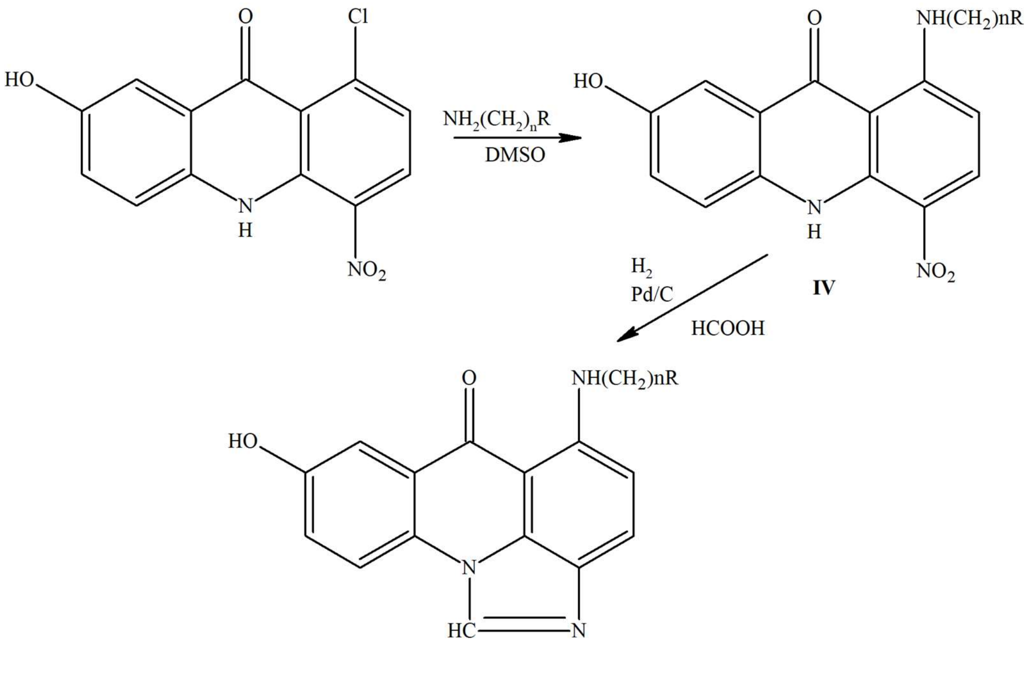

C-1311: n=2, R=$N(CH_2CH_3)_2$
Compound 1: n=2, R=NH_2

Figure S2. The strategy of C-1305 and Compound 1 synthesis.

1.3. ^{1}H NMR, ^{13}C NMR and ESI-MS Spectra for imidazo and triazoloacridinone derivatives

C-1305 analysis

^{1}H NMR

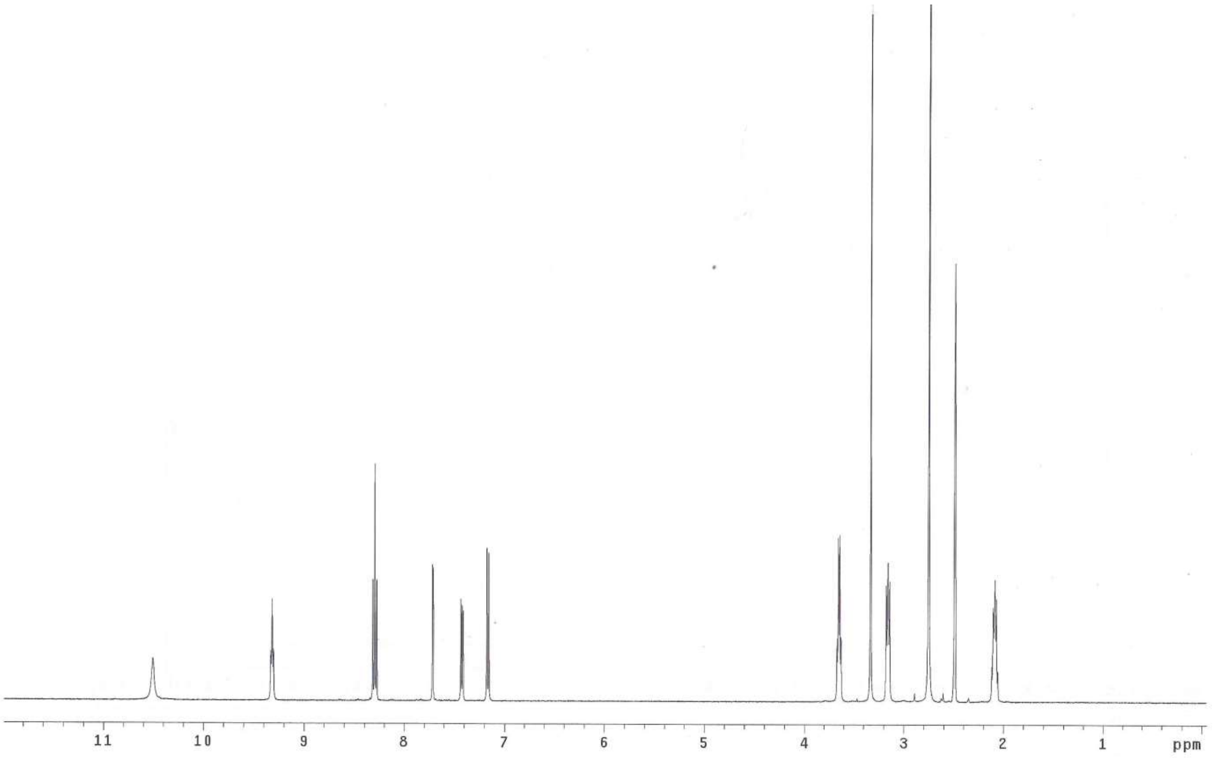

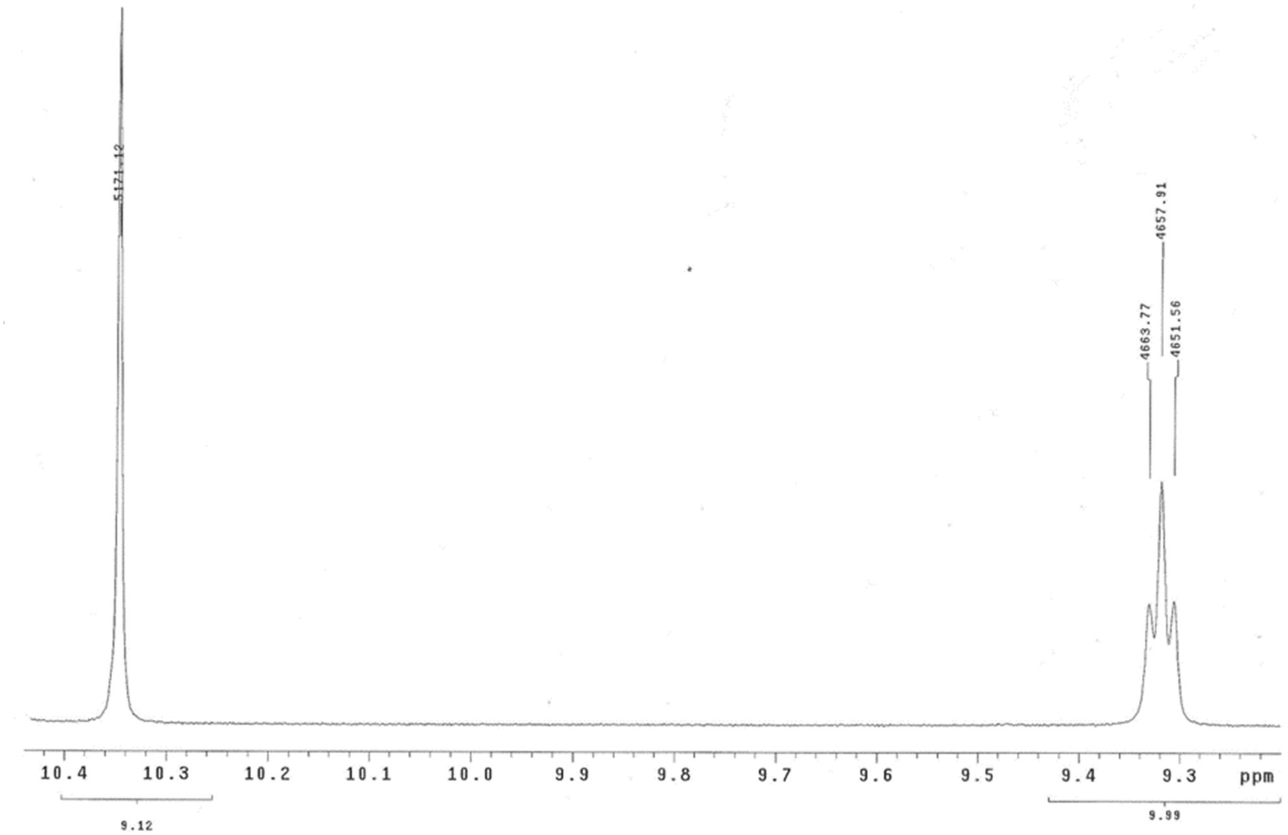
5171.12
4663.77
4657.91
4651.56
10.4 10.3 10.2 10.1 10.0 9.9 9.8 9.7 9.6 9.5 9.4 9.3 ppm
9.12
9.99

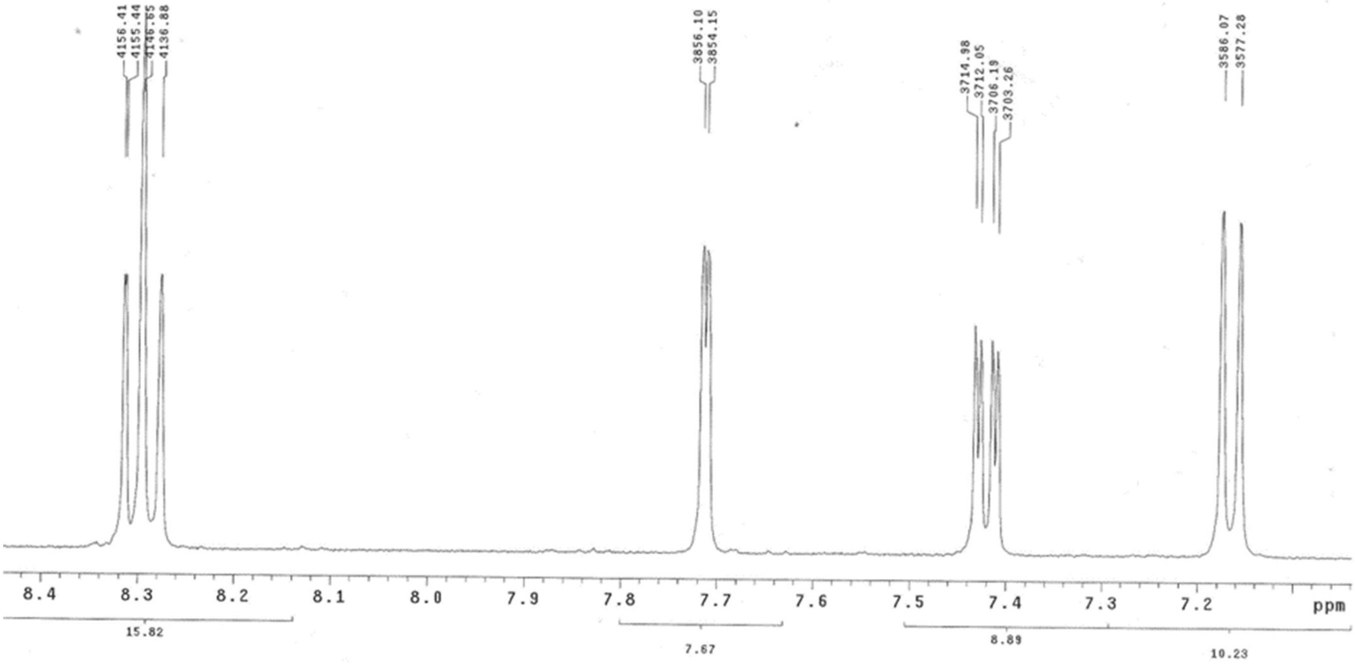
4156.41
4155.44
4146.65
4136.88
3856.10
3854.15
3714.98
3712.05
3706.19
3703.26
3586.07
3577.28
8.4 8.3 8.2 8.1 8.0 7.9 7.8 7.7 7.6 7.5 7.4 7.3 7.2 ppm
15.82
7.67
8.89
10.23

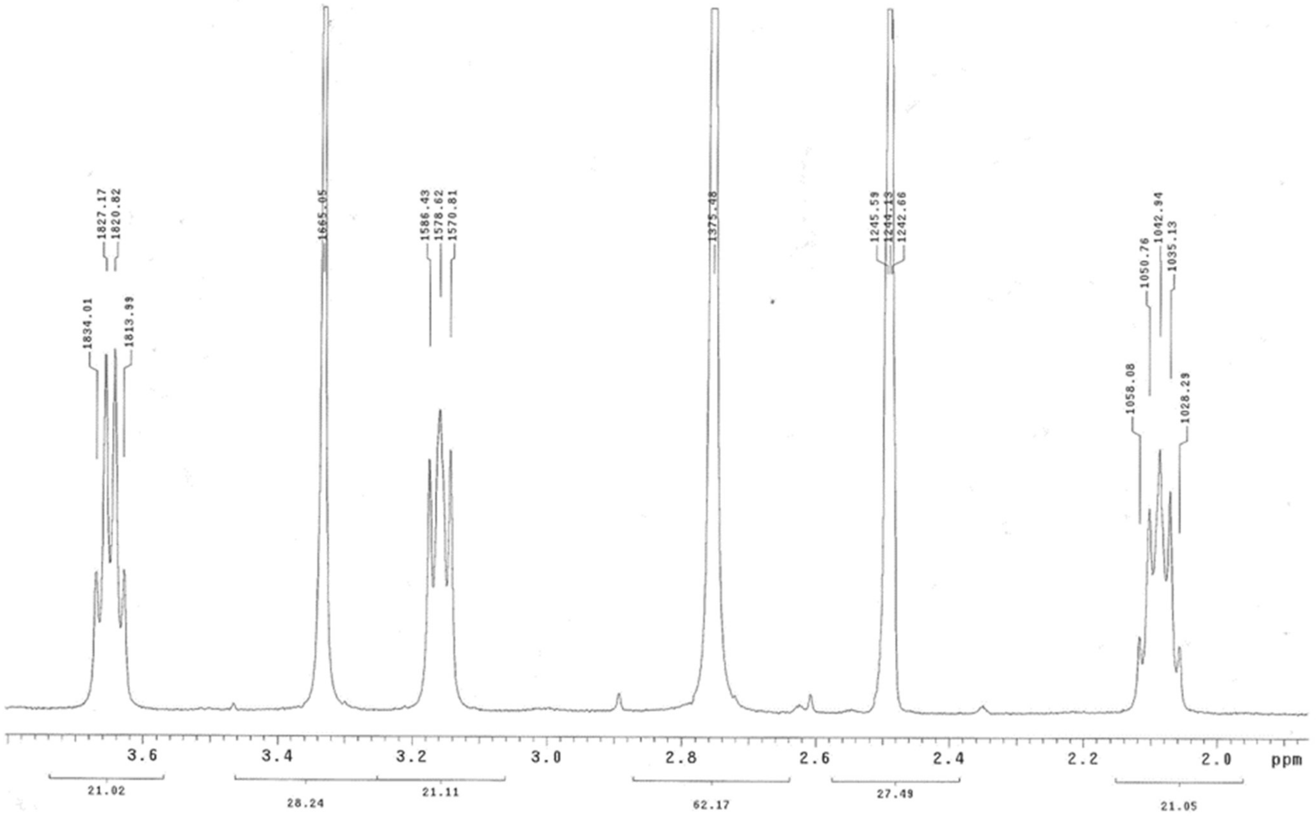
1834.01
1827.17
1820.82
1813.99
1665.05
1586.43
1578.62
1570.81
1375.48
1245.59
1244.13
1242.66
1058.08
1050.76
1042.94
1035.13
1028.29
3.6
3.4
3.2
3.0
2.8
2.6
2.4
2.2
2.0
ppm
21.02
28.24
21.11
62.17
27.49
21.05

^{13}C NMR

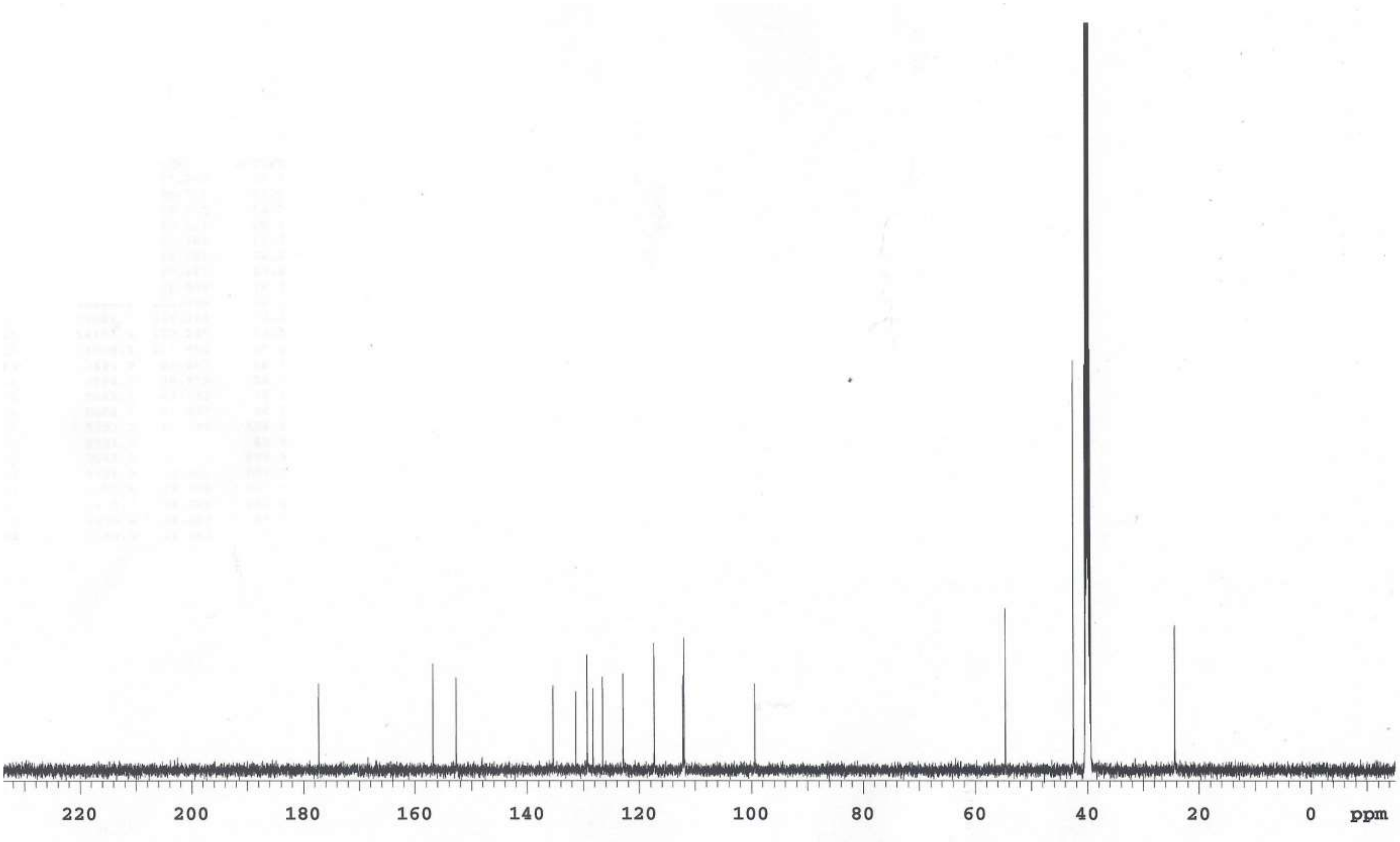
220
200
180
160
140
120
100
80
60
40
20
0
ppm

ESI-MS Spectra

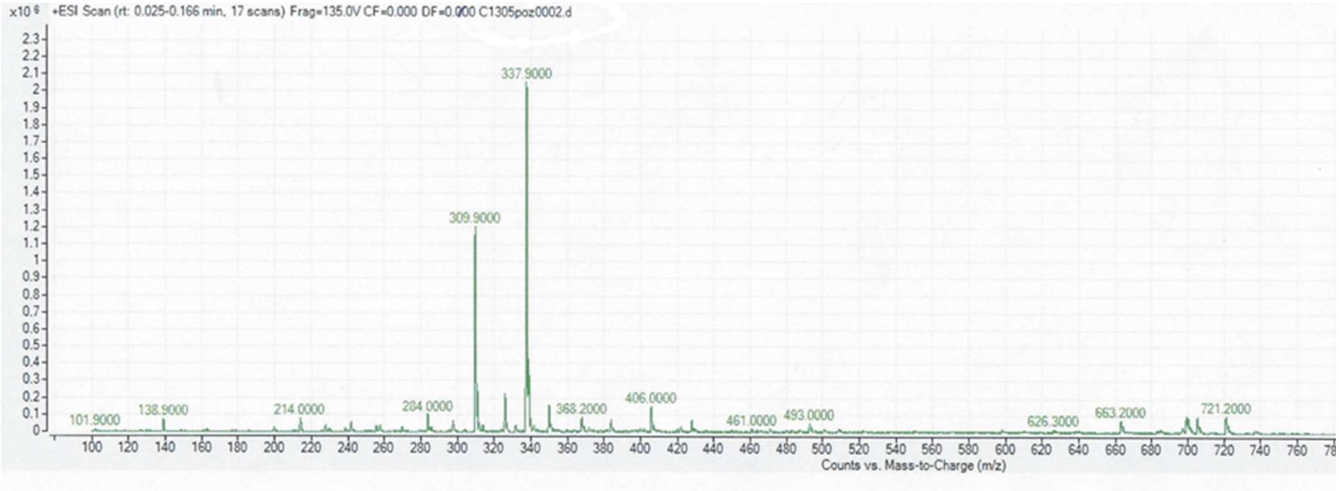

<u>C-1296 analysis</u>

¹H NMR

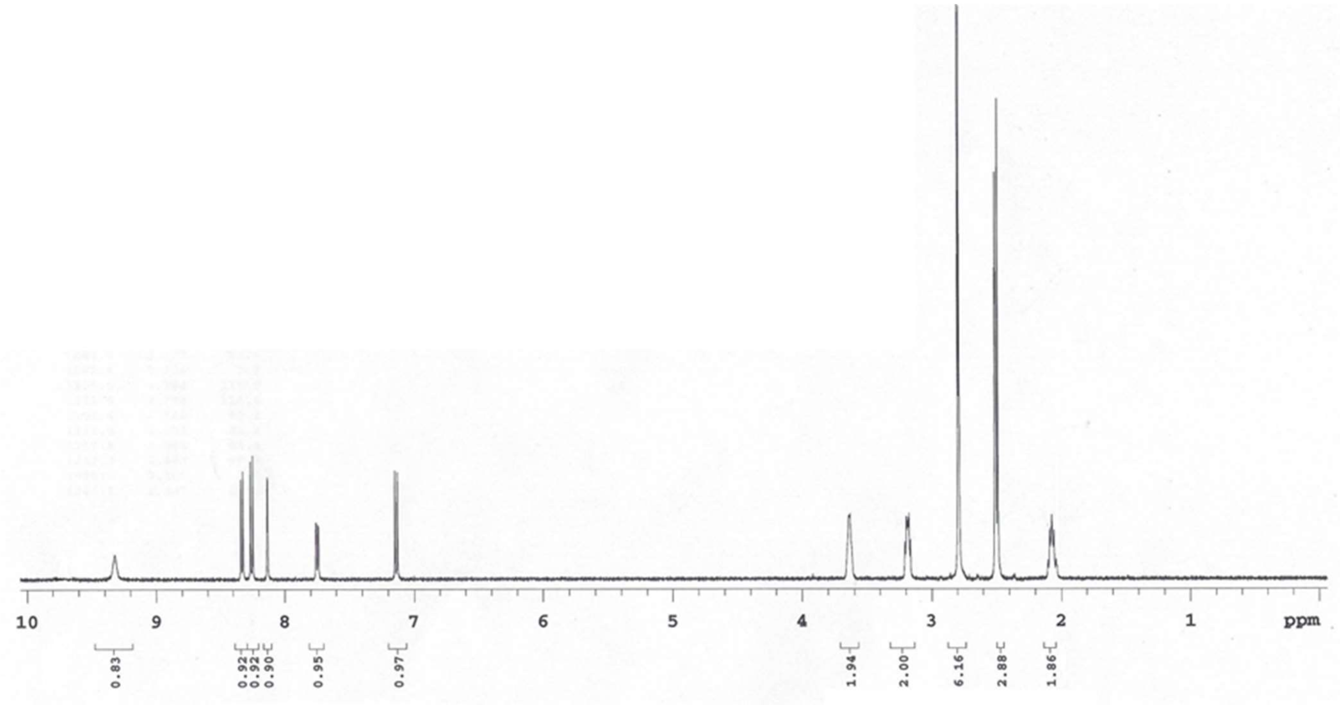

¹³C NMR

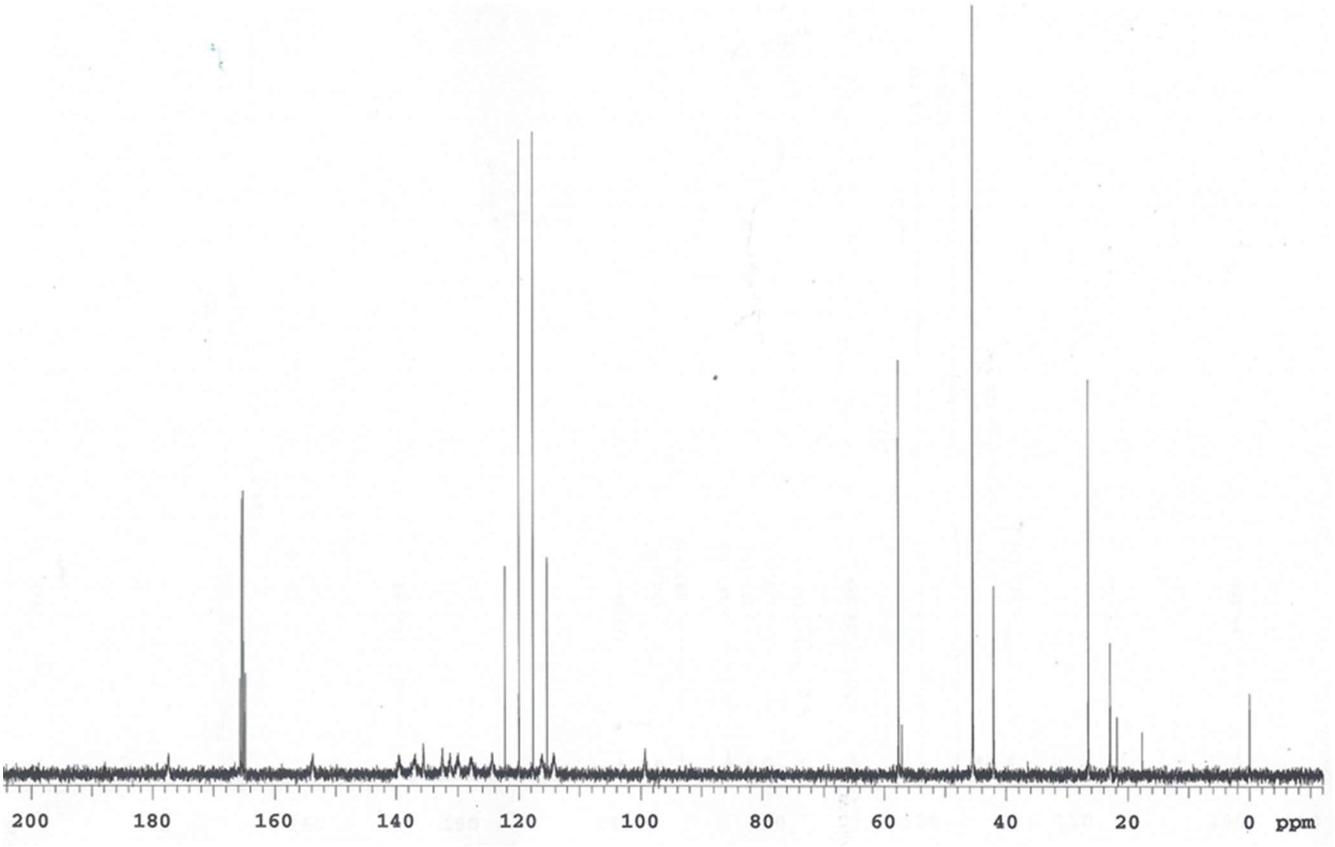

ESI-MS Spectra

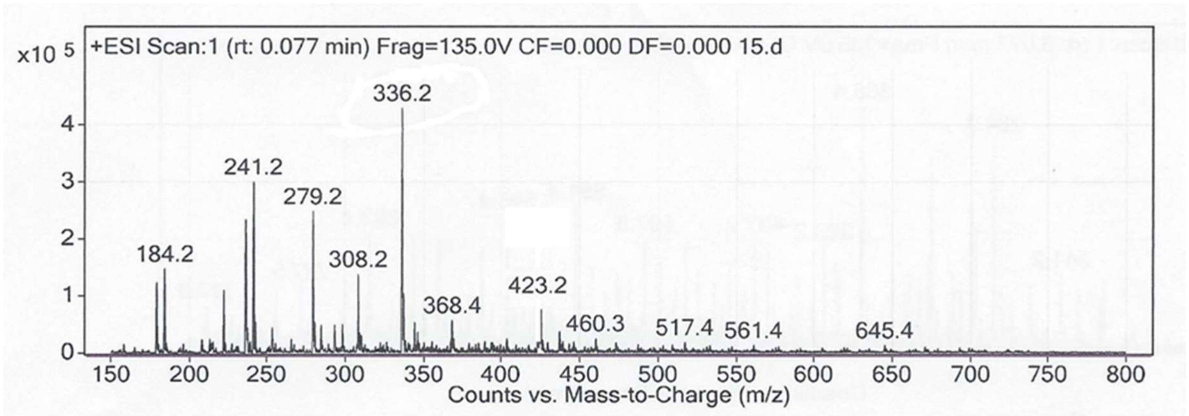

C-1410 analysis

^{1}H NMR

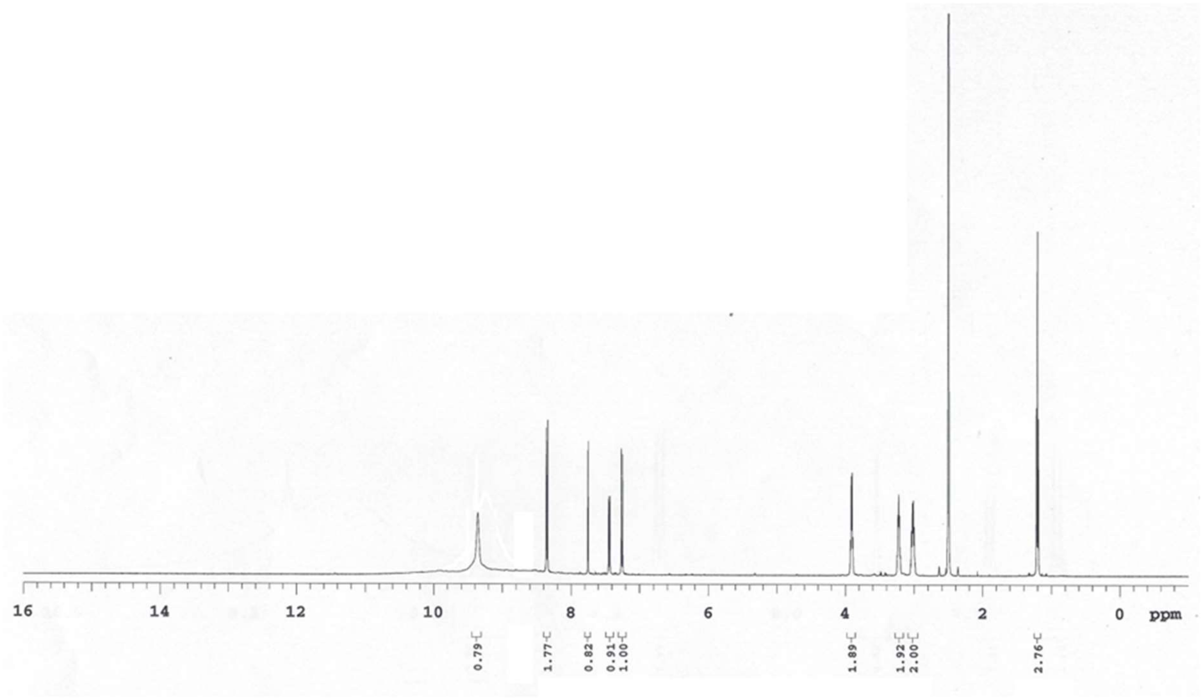

^{13}C NMR

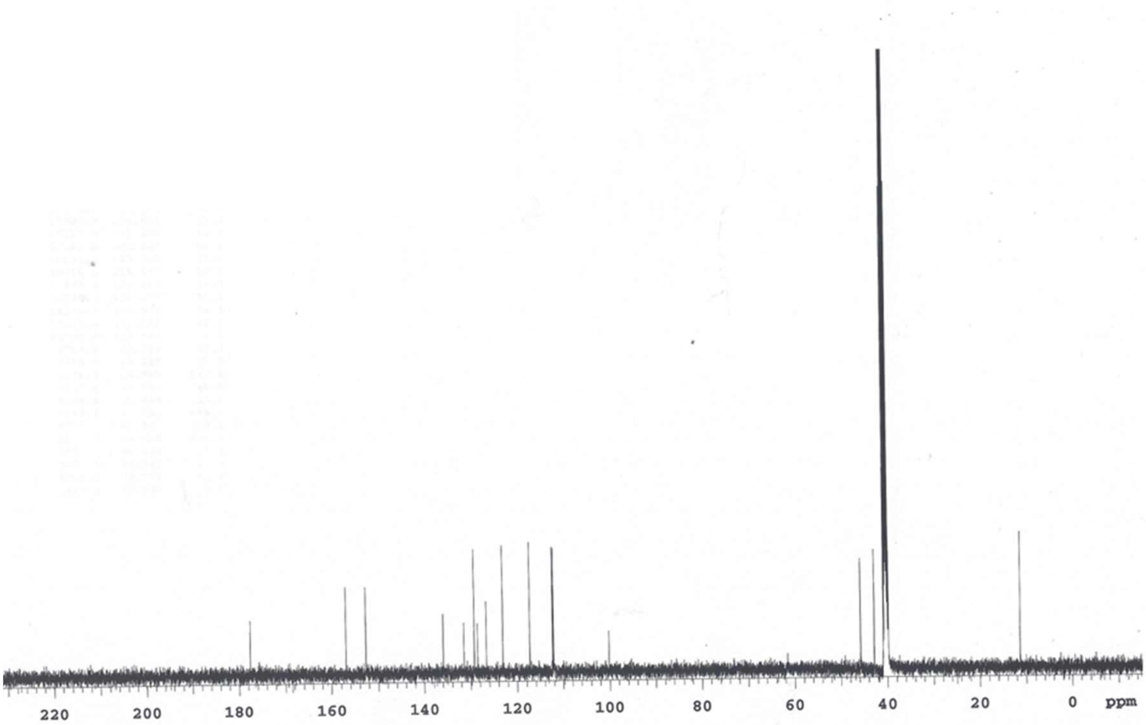

ESI-MS Spectra

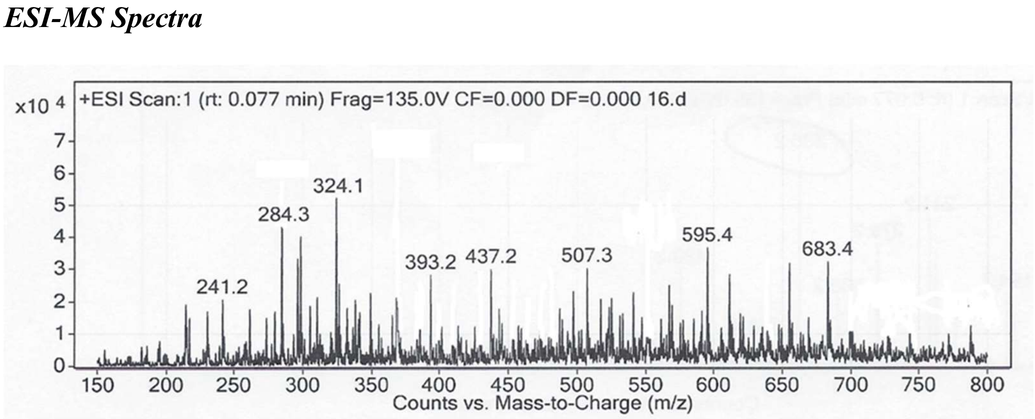

<u>**C-1311 analysis**</u>

^{1}H NMR

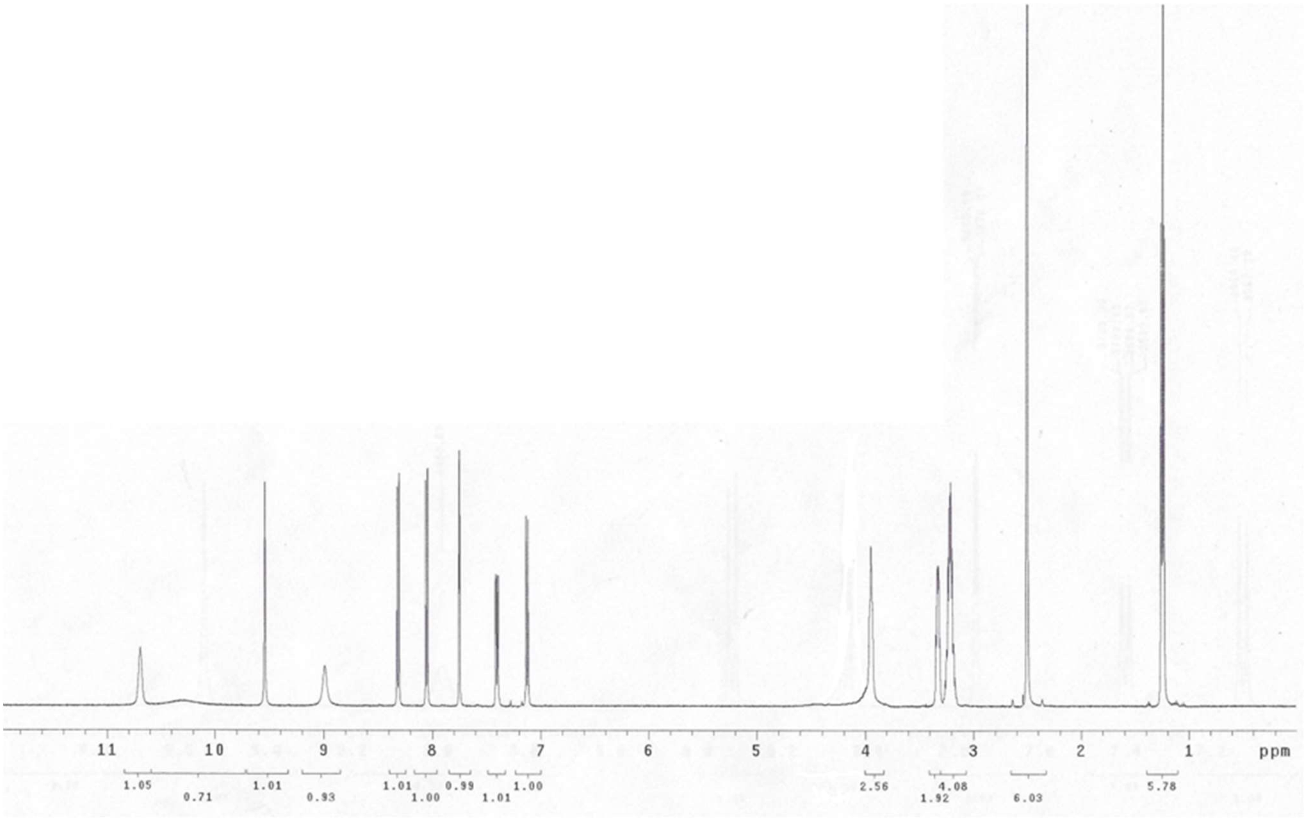

^{13}C NMR

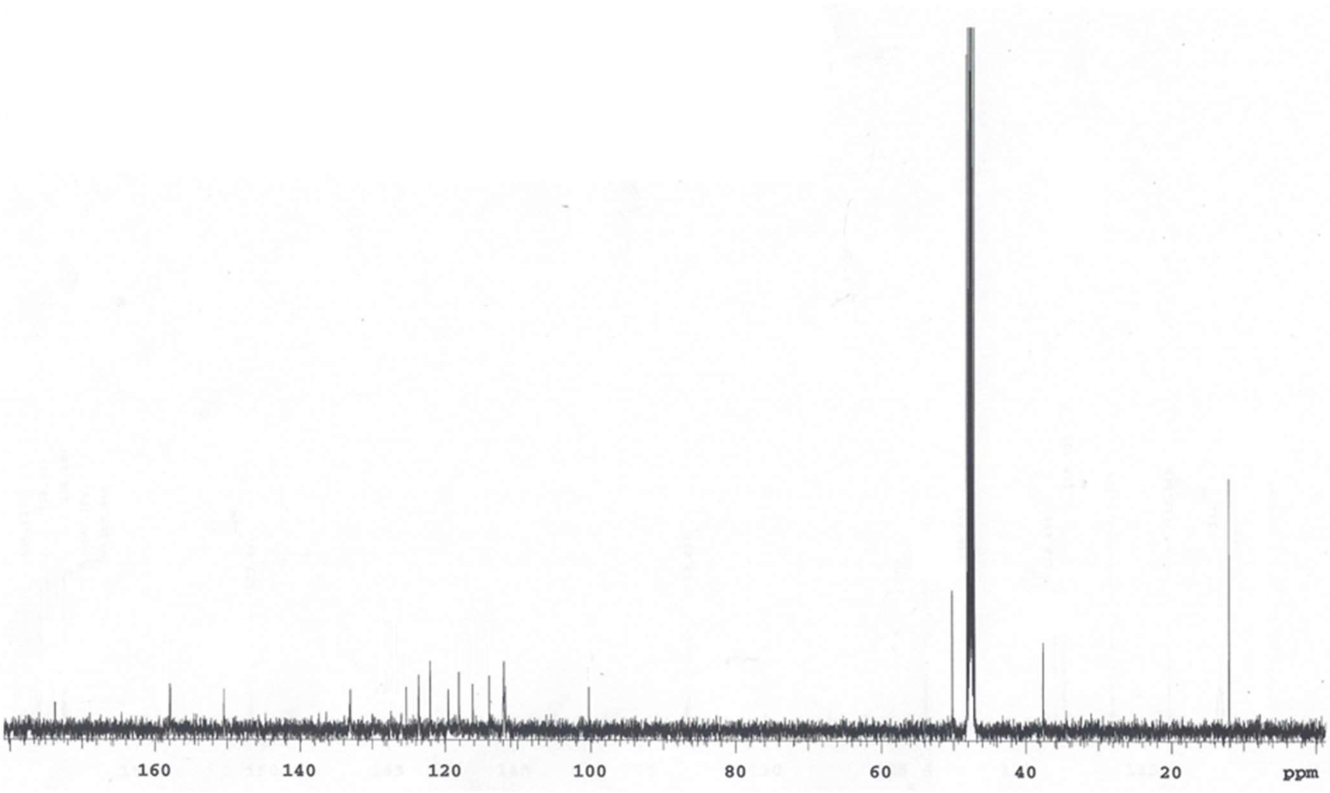

ESI-MS Spectra

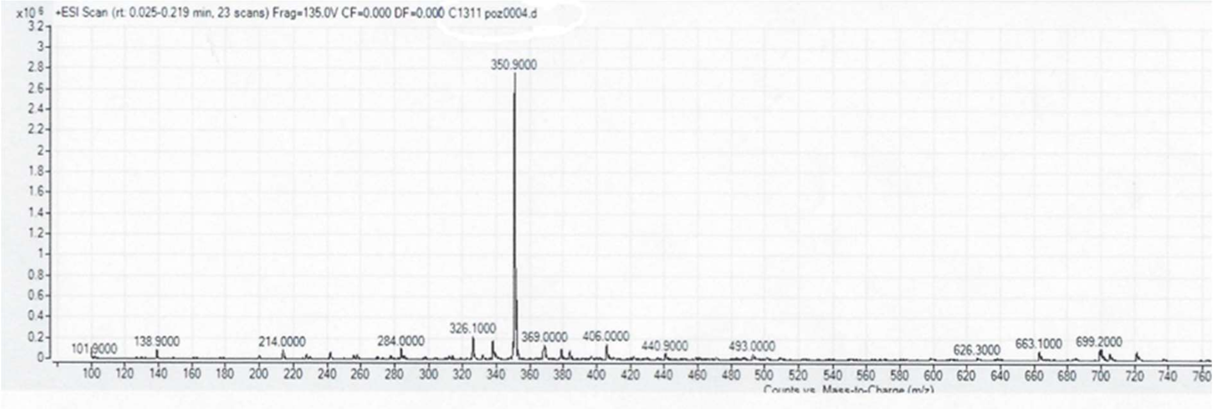

Compound 1 analysis

1H NMR

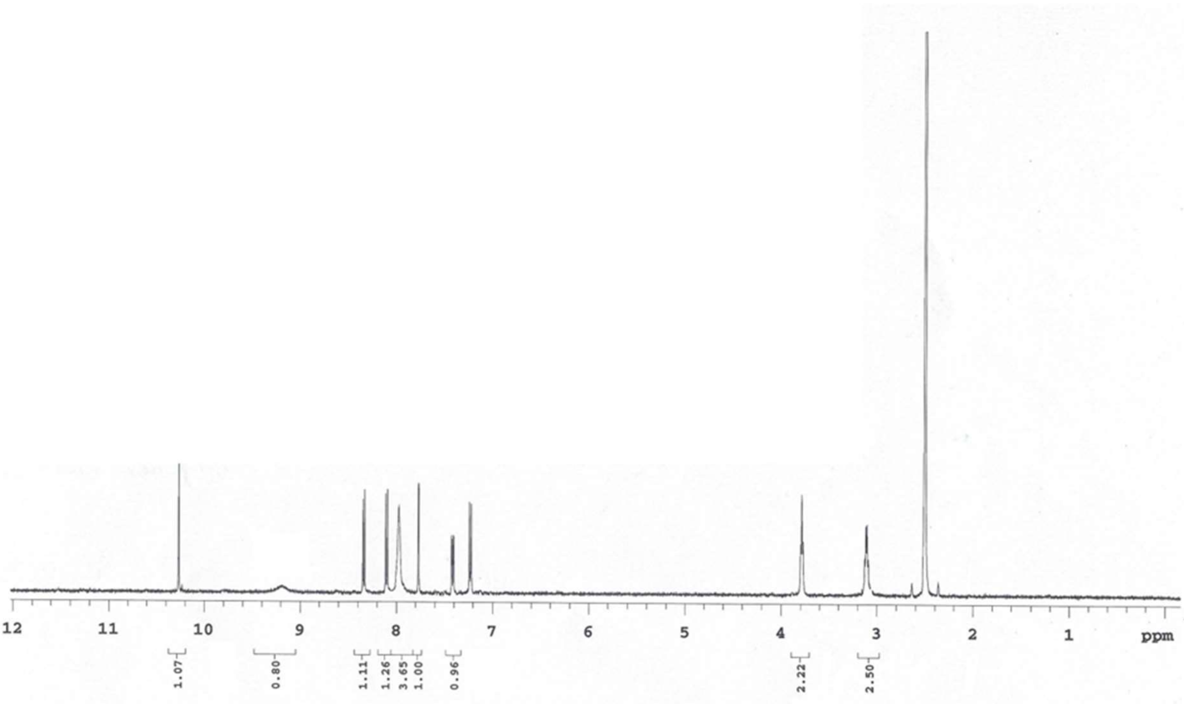

13C NMR

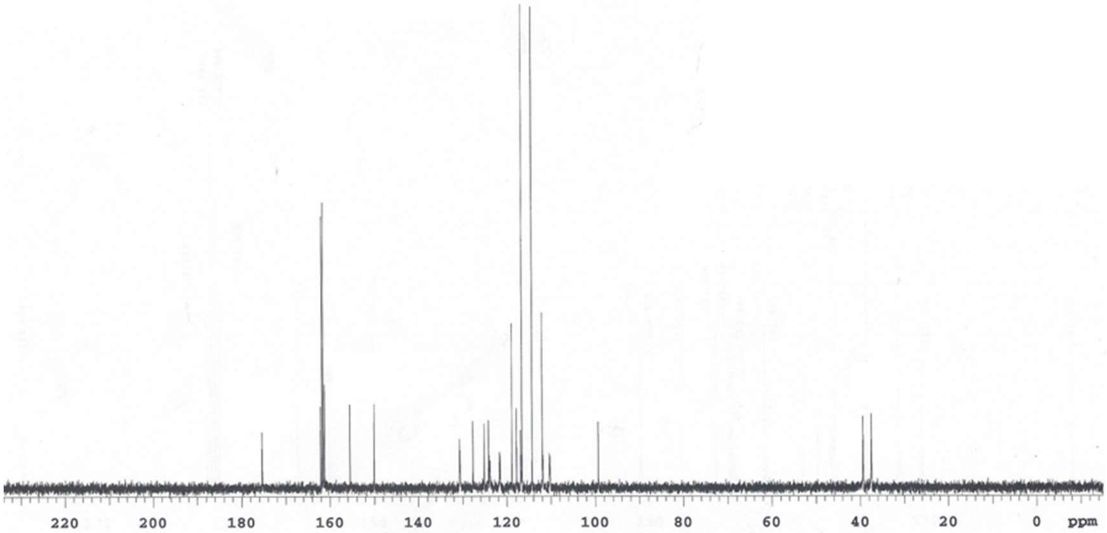

ESI-MS Spectra

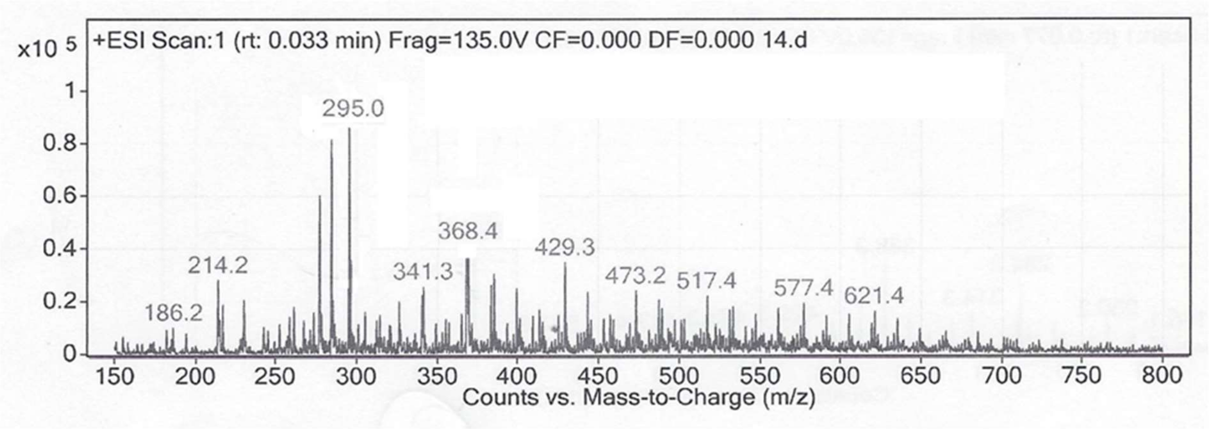

2. Compound 1-R8 synthesis and chemical analysis

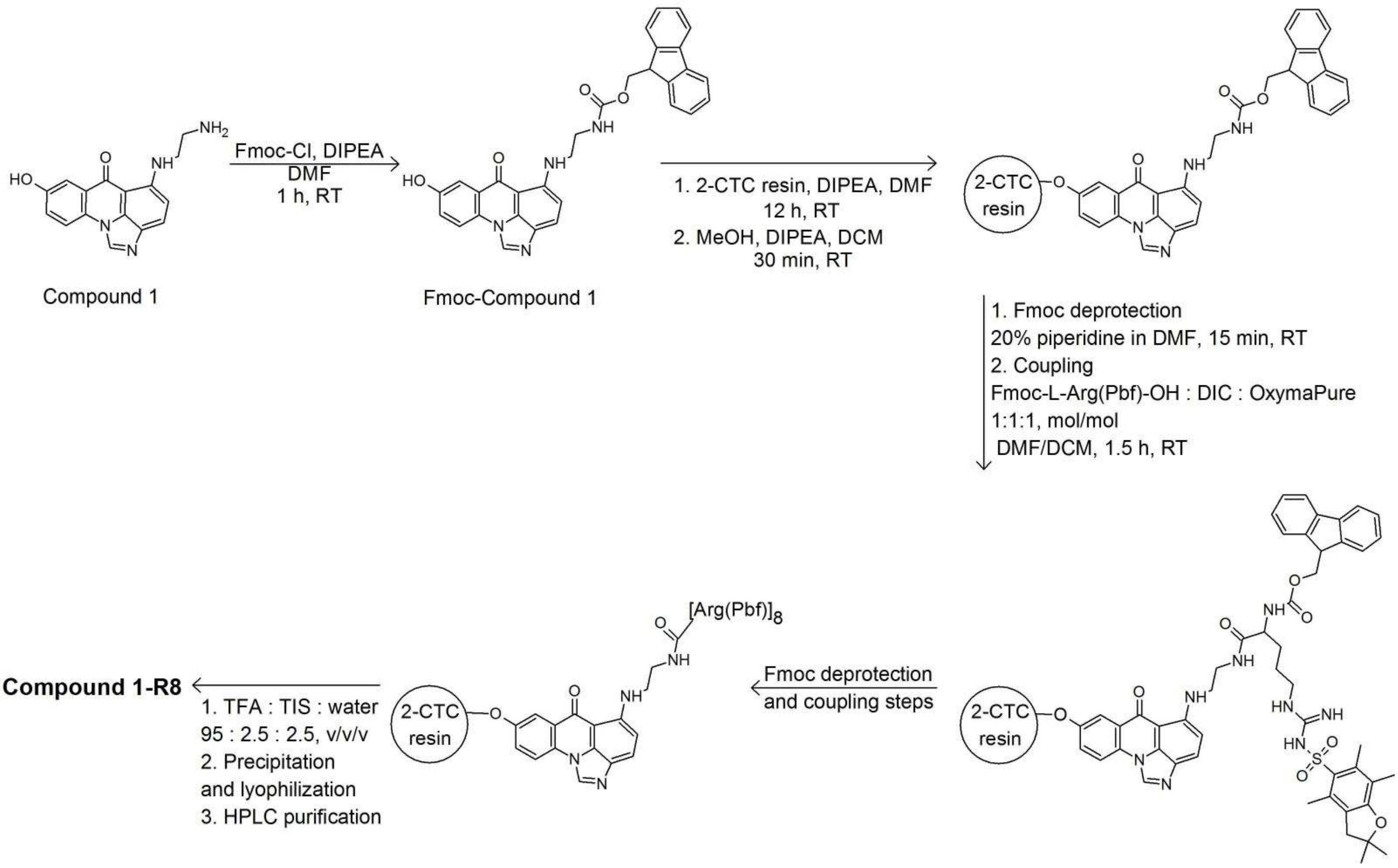

Figure S3. Scheme of Compound 1-R8 synthesis

Table S1. MS data for Compound 1-R8. Average mass: 1543.79 Da; monoisotopic mass: 1542.92 Da. Calc. mass for (M + 2H/2)+ = 772.47 Da, (M + 3H/3)+ = 515.31 Da and (M+4H/4)+ = 386.74 Da.

z	m/z calc.	m/z exp.
**1**	1543.93	-
**2**	772.47	772.43
**3**	515.31	515.69

**4**	386.74	387.13

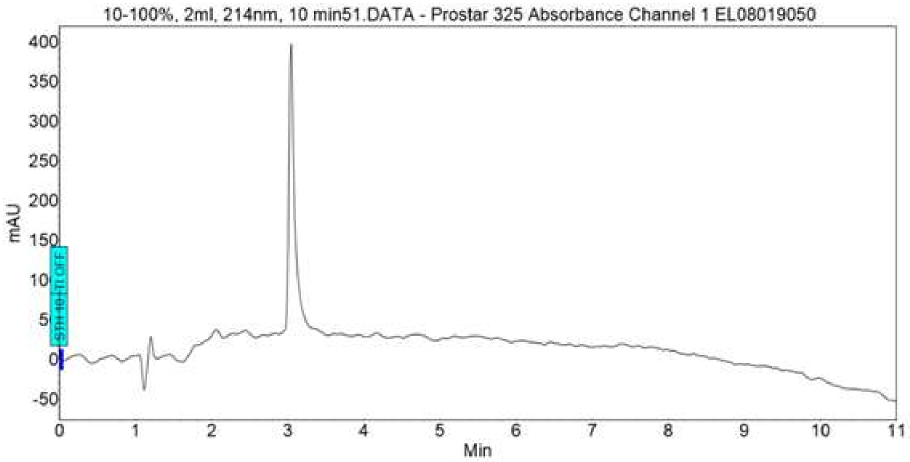

Figure S4. Analytical HPLC data of Compound 1 –R8 conjugate.

3. Compound 1-R ^{1}H NMR and COSY

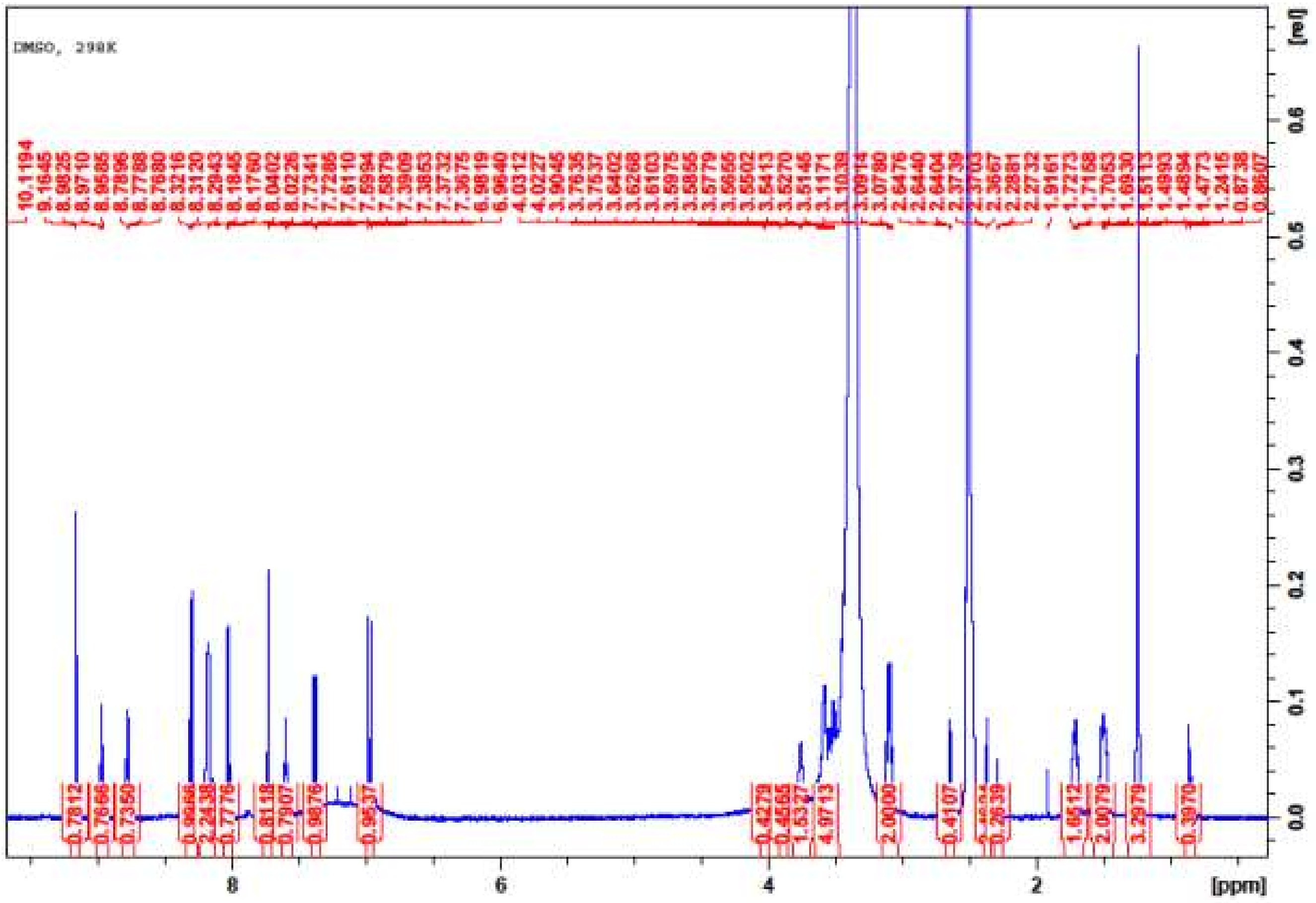

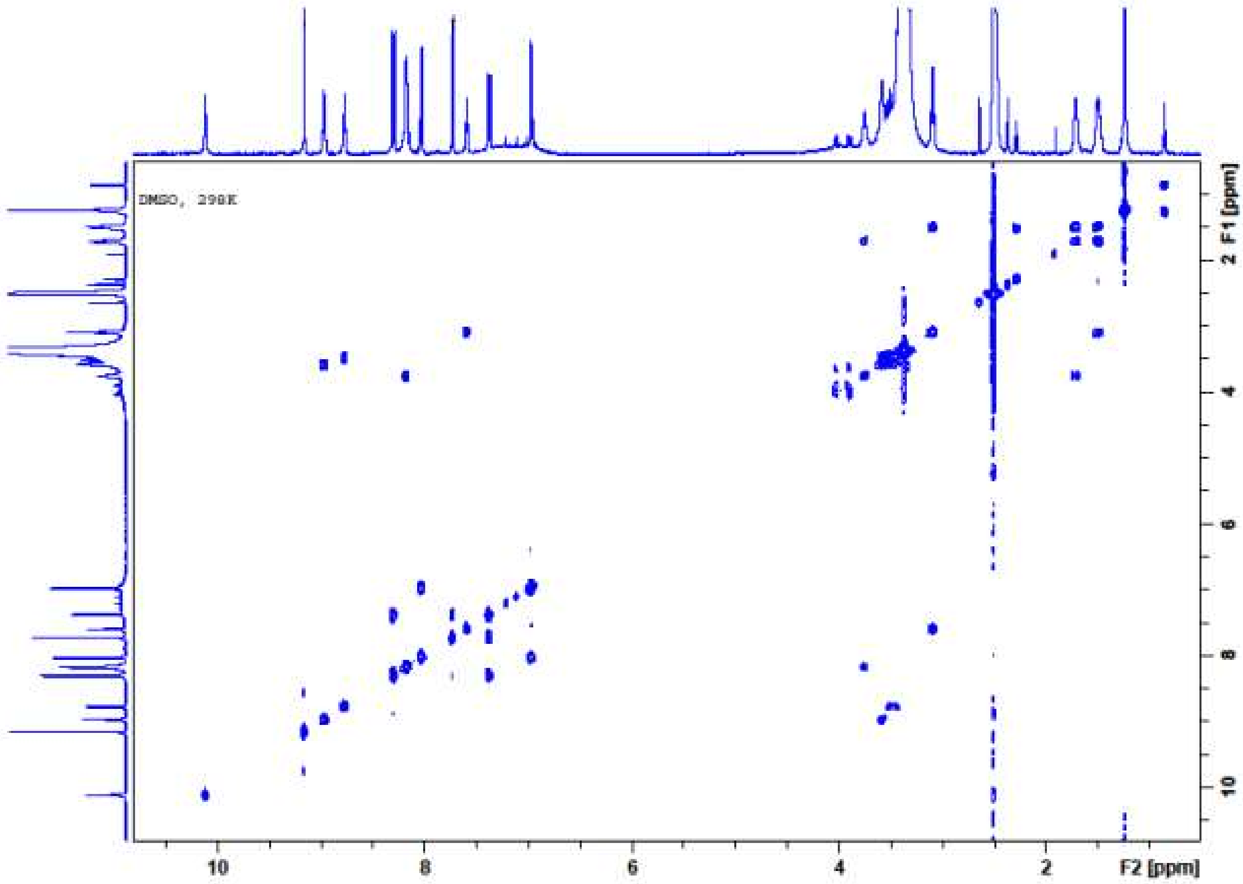
DMSO, 298K
F1 [ppm]
F2 [ppm]

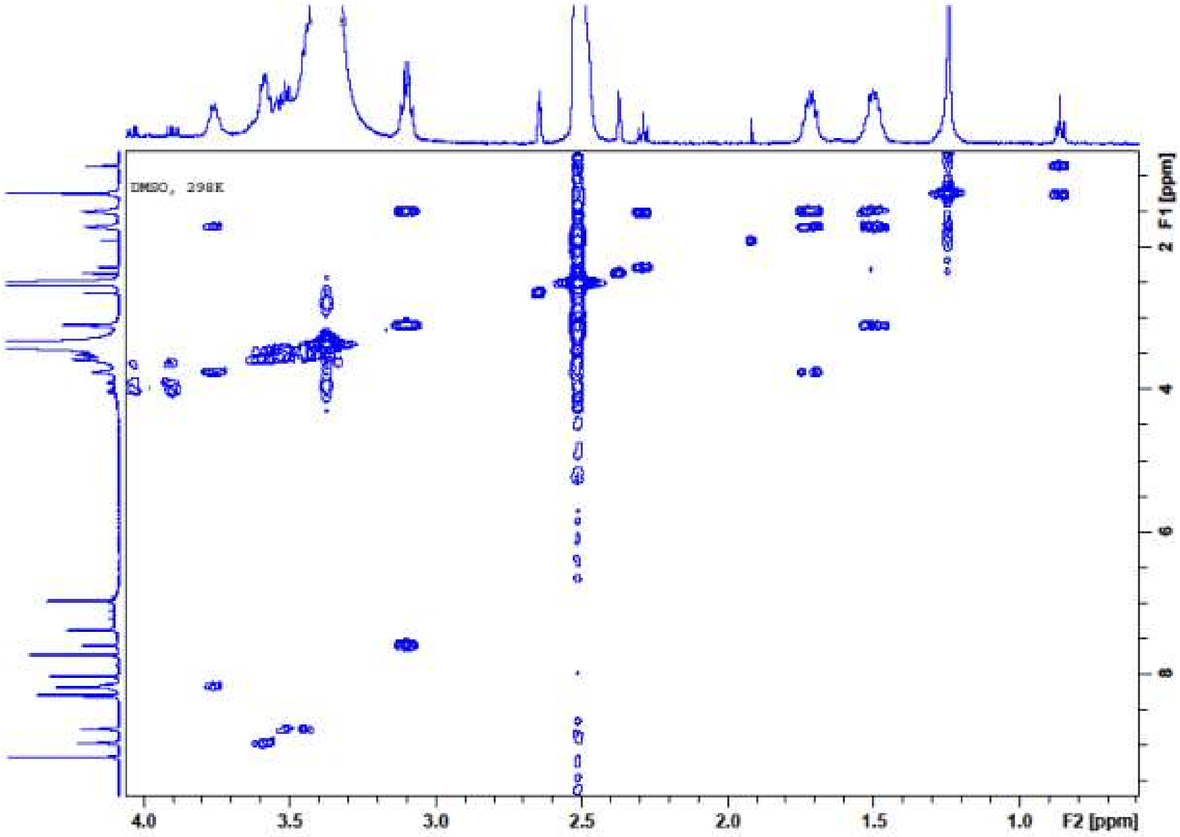
DMSO, 298K
F1 [ppm]
F2 [ppm]

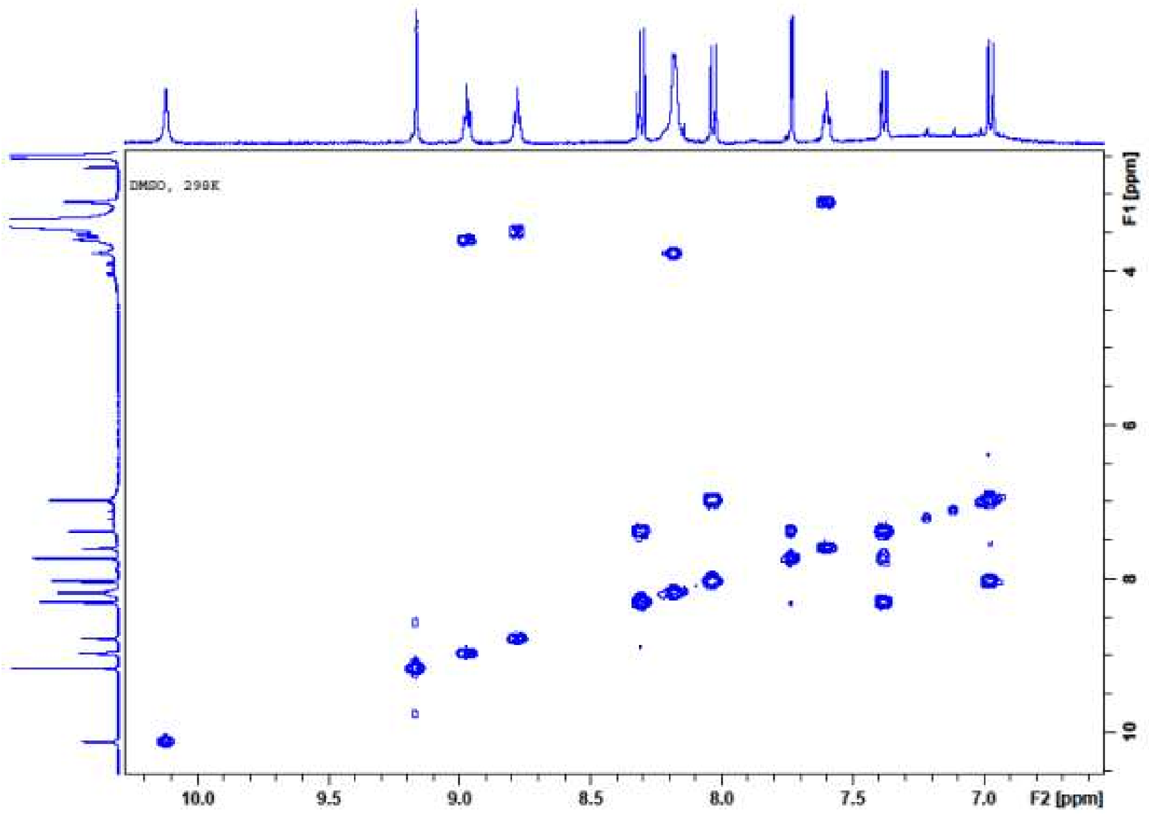
DMSO, 298K
F1 [ppm]
F2 [ppm]
10.0
9.5
9.0
8.5
8.0
7.5
7.0
4
6
8
10

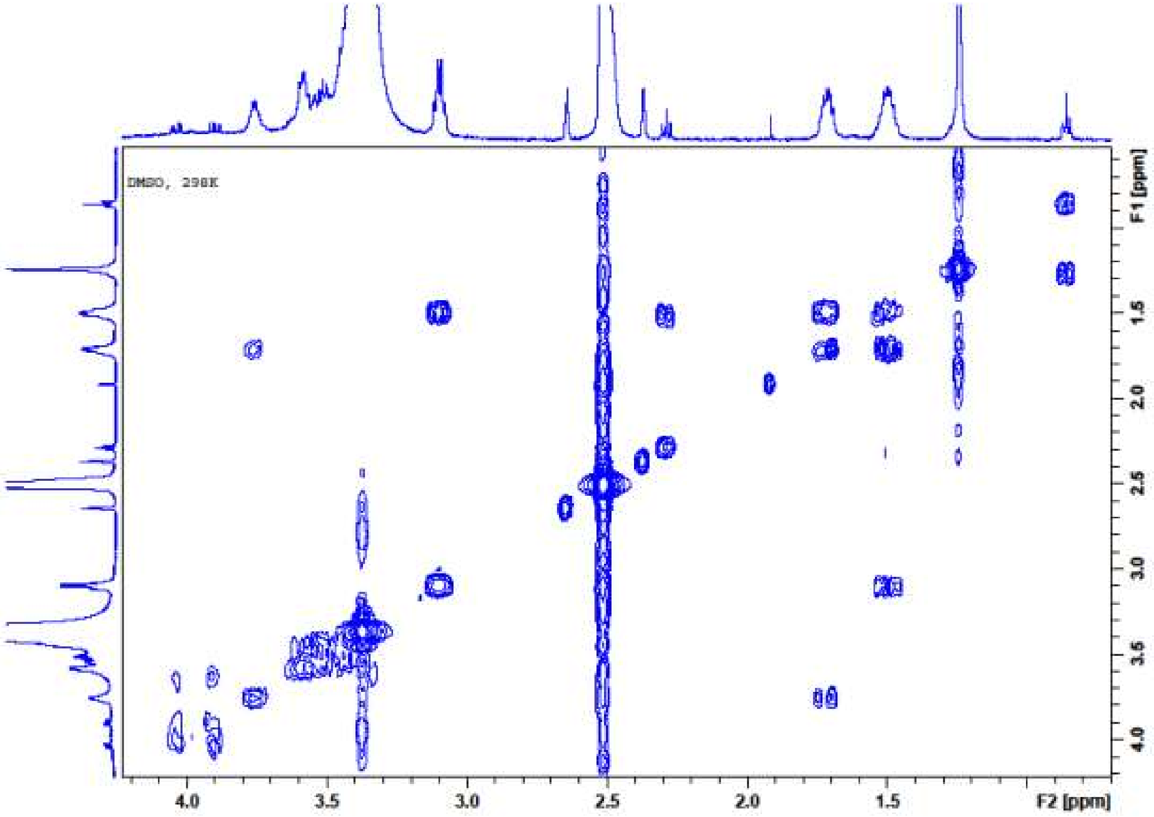
DMSO, 298K
F1 [ppm]
F2 [ppm]
4.0
3.5
3.0
2.5
2.0
1.5
1.5
2.0
2.5
3.0
3.5
4.0
